# Critical role of the gut microbiota in immune responses and cancer immunotherapy

**DOI:** 10.1186/s13045-024-01541-w

**Published:** 2024-05-14

**Authors:** Zehua Li, Weixi Xiong, Zhu Liang, Jinyu Wang, Ziyi Zeng, Damian Kołat, Xi Li, Dong Zhou, Xuewen Xu, Linyong Zhao

**Affiliations:** 1 Department of Plastic and Burn Surgery, West China Hospital, Sichuan University, Chengdu, China; 2 Chinese Academy of Medical Sciences (CAMS), CAMS Oxford Institute (COI), Nuffield Department of Medicine, University of Oxford, Oxford, England; 3 Department of Neurology, West China Hospital, Sichuan University, Chengdu, China; 4 Institute of Brain Science and Brain-Inspired Technology of West China Hospital, Sichuan University, Chengdu, China; 5 Target Discovery Institute, Center for Medicines Discovery, Nuffield Department of Medicine, University of Oxford, Oxford, England; 6 Departments of Obstetrics and Gynecology, West China Second University Hospital of Sichuan University, Chengdu, China; 7 Department of Neonatology, West China Second University Hospital of Sichuan University, Chengdu, China; 8 Department of Functional Genomics, Medical University of Lodz, Lodz, Poland; 9 Department of Biomedicine and Experimental Surgery, Medical University of Lodz, Lodz, Poland; 10 Department of Urology, Churchill Hospital, Oxford University Hospitals NHS Foundation, Oxford, UK; 11 Department of General Surgery and Gastric Cancer Center, West China Hospital, Sichuan University, Chengdu, China

**Keywords:** Cancer immunotherapy, Immune checkpoint inhibitors, Gut microbiota-derived metabolites, Immune-related adverse events, Microbiota modification

## Abstract

The gut microbiota plays a critical role in the progression of human diseases, especially cancer. In recent decades, there has been accumulating evidence of the connections between the gut microbiota and cancer immunotherapy. Therefore, understanding the functional role of the gut microbiota in regulating immune responses to cancer immunotherapy is crucial for developing precision medicine. In this review, we extract insights from state-of-the-art research to decipher the complicated crosstalk among the gut microbiota, the systemic immune system, and immunotherapy in the context of cancer. Additionally, as the gut microbiota can account for immune-related adverse events, we discuss potential interventions to minimize these adverse effects and discuss the clinical application of five microbiota-targeted strategies that precisely increase the efficacy of cancer immunotherapy. Finally, as the gut microbiota holds promising potential as a target for precision cancer immunotherapeutics, we summarize current challenges and provide a general outlook on future directions in this field.

**Supplementary Information:**

The online version contains supplementary material available at 10.1186/s13045-024-01541-w.

## Introduction

Microbes can be found throughout the human body, from external surfaces such as the conjunctiva, oral mucosa, and skin to internal surfaces such as the gastrointestinal tract and saliva. It has been estimated that trillions of bacteria, fungi, archaea, protozoa, and viruses exist throughout the body [1]. In accordance with this fact, there is also accumulating evidence that many physiological functions within the human body, including metabolism, inflammation, and the immune response, are influenced by microbes [2, 3]. Thanks to the technological boosts in large-scale sequencing over the past decade, multiple databases of the gut microbiome have been built to examine these functions(Table 1). These functions are related to the pathological processes of many human diseases, especially the development, progression, and immune evasion of cancer, as well as the modulatory effects of cancer treatments [4–7].

**Table 1:** Selected database of the gut microbiome

Official name	Description	Website
NIH Human Microbiome Project	The Human Microbiome Project (HMP) characterizes the microbial communities found at several different sites on the human body, and examines the role of these microbes in human health and disease	https://www.hmpdacc.org/health/projectdemos.php
NIH Integrative Human Microbiome Project	The Integrative Human Microbiome Project (iHMP) is the second phase of the HMP. In this phase of the program, the iHMP will create integrated longitudinal datasets of biological properties from both the microbiome and hostusing multiple "omics" technologies	https://www.hmpdacc.org/ihmp
The Animal Microbiome Database	Animal Microbiome Database (AMDB) is a database covering bacterial 16S rRNA gene profiles to assess the relationship between the gut microbiota and animal hosts. Currently, AMDB contains 10,478 bacterial taxa from 467 animal species	http://leb.snu.ac.kr/amdb
Peryton	Peryton constitutes a novel resource of experimentally supported microbe-disease associations, currently linking 43 diseases and 1,396 microorganisms	https://dianalab.e-ce.uth.gr/peryton
gutMDisorder	GutMDisorder, a manually curated database, aims at providing a comprehensive resource of dysbiosis of the gut microbiota in disorders and interventions, derived from manual literature extraction and raw data reprocessing	http://bio-annotation.cn/gutMDisorder
Type Strains Genome Database	The type strain sequencing project is an international coordinated effort to close current gaps in the genomic maps of microbes and hence to comprehensively decipher the characteristics of microorganisms through deep mining of the genomic data	http://gctype.wdcm.org
The Integrated Microbial Genomes system	The Integrated Microbial Genomes (IMG) system serves as a community resource for analysis and annotation of genome and metagenome datasets in a comprehensive comparative context	https://img.jgi.doe.gov/m
The SILVA ribosomal RNA gene database project	SILVA provides comprehensive, quality checked and regularly updated datasets of aligned small (16S/18S, SSU) and large subunit (23S/28S, LSU) ribosomal RNA sequences for all three domains of life (*Bacteria*, *Archaea* and *Eukarya*)	http://www.arb-silva.de
National Microbiome Initiative	The National Microbiome Initiative aims at supporting interdisciplinary research to answer fundamental questions about microbiomes and developing platform technologies that will generate insights of microbiomes and enhance access to microbiome data	–
The Integrated Gene Catalog	The integrated gene catalog is comprised of 9,879,896 genes including samples from the MetaHit project. This expanded catalog should facilitate characterization of metagenomic, metatranscriptomic and metaproteomic data from the gut microbiome to understand its variation in human health and disease	–
MetaGenoPolis	MetaGenoPolis offers a State-of-the-art equipment for biobanking, DNA extraction, sequencing, screening, bioinformatics, and data visualization. It owns 100 publications in metagenomics	https://mgps.eu
The Michigan Microbiome Project	The Michigan Microbiome Project (MMP) focused on studying how these microbes originate, function, and evolve. This is a repository of 16S gene sequence surveys, transcriptomes, metagenomes, metabolomes, and information about isolated strains	https://microbe.med.umich.edu
Home Microbiome Project	The Home Microbiome Project followed 7 families over the course of 6 weeks. The participants in the study swabbed their hands, feet and noses daily to collect a sample of the microbial populations living in and on them	https://homemicrobiome.com

The essential properties of the gut microbiota, such as its stability, resilience, and diversity, need to be discussed, given its importance in human health [8]. The gut microbial community can be stable for years in healthy adults; thus, the microbiota has high stability. Homeostasis of the gut microbiota is maintained through negative feedback mechanisms [9]. The gut microbiota is often highly resilient to perturbations, thus allowing a host to maintain key species for long periods. However, understanding the resilience of this complex gut ecosystem is still challenging because the threshold for transitions of the gut microbiota to different states is only beginning to be determined [10, 11]. Microbial interactions ranging from mutualism and commensalism to competition and amensalism and the symbiotic relationship between microbes and their host can be considered essential factors in shaping gut stability and resilience of the gut microbiota [12]. With the recent advent of high-throughput sequencing, the diversity of the gut microbiota has been revealed at both the species and functional levels [13]. Functional screening by shotgun metagenomics contributes significantly to understanding the functional diversity of the gut microbiome. As more complementary “omics” datasets become available, functional variation in the gut microbiota in response to disease, diet, or other factors may be discovered [14]. For studies focusing on the diversity of the gut microbiota, a key challenge is understanding functional redundancy (i.e., which community species have similar functional niches and can substitute for one another). Funtional redundancy is also a critical aspect for conferring stability and resilience to the gut microbiota [15].

The gut microbiota has been shown to play critical roles in maintaining intestinal barrier integrity and homeostasis. The composition of the gut microbiome is under the surveillance of the intestinal immune system. Inflammation caused by an imbalance between commensal and pathogenic microbes can lead to intestinal and even systemic diseases [16]. In terms of the mutually beneficial symbiotic ecosystem between the gut microbiota and the host, the host offers habitats and nutrients in the gut, while the microbes support the maintenance of lipid and glucose metabolism and the maturation of the intestinal immune system by providing microbiome-derived metabolites [17]. For instance, short-chain fatty acids (SCFAs), including acetic acid, butyric acid, and propionic acid, are essential energy sources for gut microbes and perform diverse regulatory functions related to host physiology and immunity [18]. Trimethylamine N-oxide (TMAO), which is a molecule generated from gut microbial metabolism, is also associated with host immunity [19].

Current research on the relationship between cancer and microbes has mostly focused on the gut microbiota and demonstrated a complicated interaction between the gut microbiota and the immune system; this interaction was evaluated by determining the composition of the gut microbiota [20]. For example, observations of developmental defects in germ-free (GF) mice suggest that systemic immune function may be impaired in the absence of the gut microbiota [21]. Moreover, the gut microbiota and its metabolites have been proposed to be critical factors involved in modulating the efficacy and toxicity of cancer immunotherapy. A landmark example was presented by Sivan et al. [22], who first reported the complicated crosstalk between the gut microbiota and programmed cell death protein-1 (PD-1)/PD-1-ligand 1 (PD-L1) blockade.

Consistent with the demonstrated relationships between the gut microbes and immune responses, many in vitro and in vivo studies have also noted a promising approach for optimizing the therapeutic outcomes of cancer immunotherapy: manipulating the composition of the gut microbiota [23, 24]. However, although the concept of using the gut microbiota as a tool for precision medicine has developed rapidly over the last decade [25], the number of published studies exploring practical interventions to modify the gut microbiota is rather limited and unspecific. In this review, we will discuss five commonly explored interventions that have had relatively strong impacts on the therapeutic outcomes of cancer immunotherapy, namely, fecal microbiota transplantation (FMT), diet, probiotics, prebiotics, and engineered microbial products. Compared with the other four methods, FMT is a well-established clinical approach recommended by the FDA for modulation of the gut microbiota. The gut microbes from a healthy host are transplanted to recover microbial homeostasis in the recipient. However, the research has been restricted to correlation relationships rather than causality, and outlining the future direction of clinical applications utilizing the gut microbiota is challenging. With multiomics tools and synthetic biology, we can now explore the exact mechanism underlying gut microbiota modification in cancer immunotherapy. Here, we will also provide evidence to support the incorporation of gut microbiota modification in immunotherapy while acknowledging the challenges in this rapidly developing field.

## The interplay between the immune system and the gut microbiota

Gut microbiota symbiosis plays a multifaceted role in shaping the immune responses of the human host [26, 27]. This complicated crosstalk allows for the normal functioning of immune tolerance and immunosurveillance, which recognizes and eliminates opportunistic bacteria to prevent potential infection. The critical role of the gut microbiota in the formation of a fully functional immune system was identified in GF animals [28]. As a go-to animal model for bacteria-host interactions, GF animals display distinct features in the gut, including an immature mucus system, unformed gut-associated lymphoid tissues, and a reduced number of immune cells [29–33]. Here, we summarize the current views on how the gut microbiota influences various components of the systemic immune system. We roughly divided the following discussion into three parts: non-gastrointestinal (GI) tract lymphoid organs, the innate immune system, and adaptive immune system components in the GI tract. Specifically, we summarize the interactions between immune cells and gut microbiota (Table 2).

**Table 2:** Interactions between immune cells and gut microbiota

Immune cell types	Crosstalk with the gut microbiota	Ref
Macrophages	1. *B. fragilis* enhances their phagocytic functions 2. The gut microbiota supports the interaction between macrophages and other immune cells 3. The microbial products inhibit the release of inflammatory factors, cause macrophage metabolism alteration and induce potent Th cell response	85–89
DCs	1. The gut microbiota controls the basal state of DCs 2. The microbiota-derived signals could promote intestinal homeostasis by affecting the secretion of DCs	93–95
NK cells	1. The gut microbiota controls the innate mucosal defense provided by NK cells 2. The crosstalk between NK cells and the gut microbiome mediated by specific transcription factors could promote intestinal homeostasis	97–102
B cells	1. B cells assist in maintaining a noninflammatory host-microbe relationship by secreting immunoglobulins and cytokines 2. The gut microbiota and its metabolites could promote B cell maturation, differentiation and enhance specific IgA antibody response. This process could be influenced by IgA, cytokines, or even B cells themselves to form a symbiotic regulatory loop	106–117
CD8 + T cells	1. CTLs can be activated in TME by the intestinal microbiota and its metabolites 2. Microbial dysbiosis exacerbates can cause CD8 + T cell exhaustion 3. Butyrate could exert a direct antagonistic influence on the HDACs of CTLs and Tc17 cells and activate CD8 + T cells to differentiate into memory cells	119–125
Th cells	1. The gut microbe-derived metabolites regulate Th1 and Th2 cell functions 2. The gut microbiota modulates the activation, plasticity, and differentiation of Th17 cells 3. Different gut microbes-derived metabolites modulate Th17 cell immunological function and differentiation 4. Different diets have also shown complicated impacts on Th17 cells via alterations in the gut microbiota	130–133, 142–155
Tfh cells	1. The gut microbiota and its metabolites can induce the differentiation of Tfh cells, facilitate systemic Tfh cell responses, and regulate Tfh cell abundance 2. The gut microbiota exhibits the potential to influence systemic Tfr cells and induce the differentiation of Tfr cells	157–165
Treg cells	1. The gut microbial signals could modulate the development of Treg cells and their IL-10 expression 2. The SCFAs have been demonstrated to regulate the size and function of the Treg cell pool	171–178

### Lymphoid organs

**Fig. 1:**
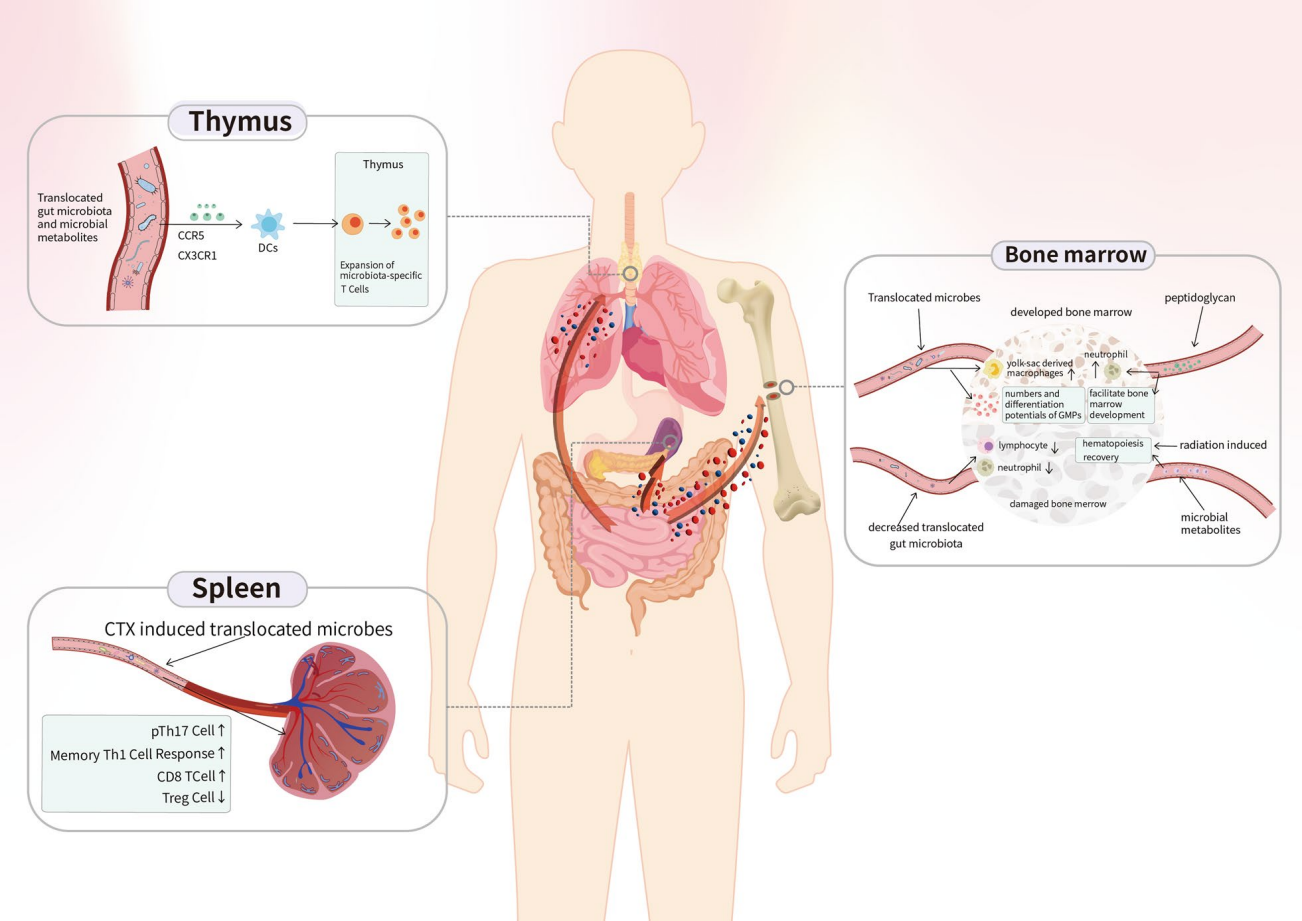
The interplay between the immune system and the gut microbiota in non-GI tract lymphoid organs. The gut microbiota and its metabolites influence the development of host bone marrow and thymus. For instance, SCFAs are capable of facilitating hematopoiesis recovery of bone marrow after radiation damage.The gut microbiota also induce the translocation of selected bacteria into and stimulate immunocytes and immune responses of the spleen after CTX treatment

Regarding the interplay of non-GI tract lymphoid organs with the gut microbiome, several studies have revealed immunological modulation by microbes in the thymus, bone marrow, and spleen. Initial clinical evidence showed an association between primary lymphoid organs and the gut microbiota in patients with hematologic malignancies [34, 35]. This association was further validated with mouse models by Staffas et al. [36], where depletion of the gut microbiota led to significant reductions in lymphocyte and neutrophil counts. Moreover, metabolites such as SCFAs can facilitate the recovery of hematopoiesis in bone marrow after radiation damage [37]. The developed bone marrow can work together with translocated gut microbiota to drive the expansion of yolk sac-derived macrophages, increase the number of granulocytes and monocyte progenitors, and promote their differentiation [38]. In addition, bone marrow development can also be affected by peptidoglycans, which modulate neutrophil function [39]. In the thymus, studies have demonstrated that recolonization of the gut microbiota drives the thymic expansion of T cells. Specifically, the gut microbiota is trafficked to the thymus in a CX3CR1- and CCR5-dependent manner by intestinal CX3CR1 DCs, which assist in inducing the expansion of microbiota-specific T cells [40]. Researchers have demonstrated that cyclophosphamide (CTX) induces the translocation of selected bacteria into the spleen, followed by the stimulation of a specific subset of “pathogenic” helper T (Th) 17 cells, which generate memory Th1 immune responses and increase the CD8 + /Regulatory T(Treg) cell ratio [41, 42] (Fig. 1).

### Antimicrobial peptides (AMPs)

**Fig. 2:**
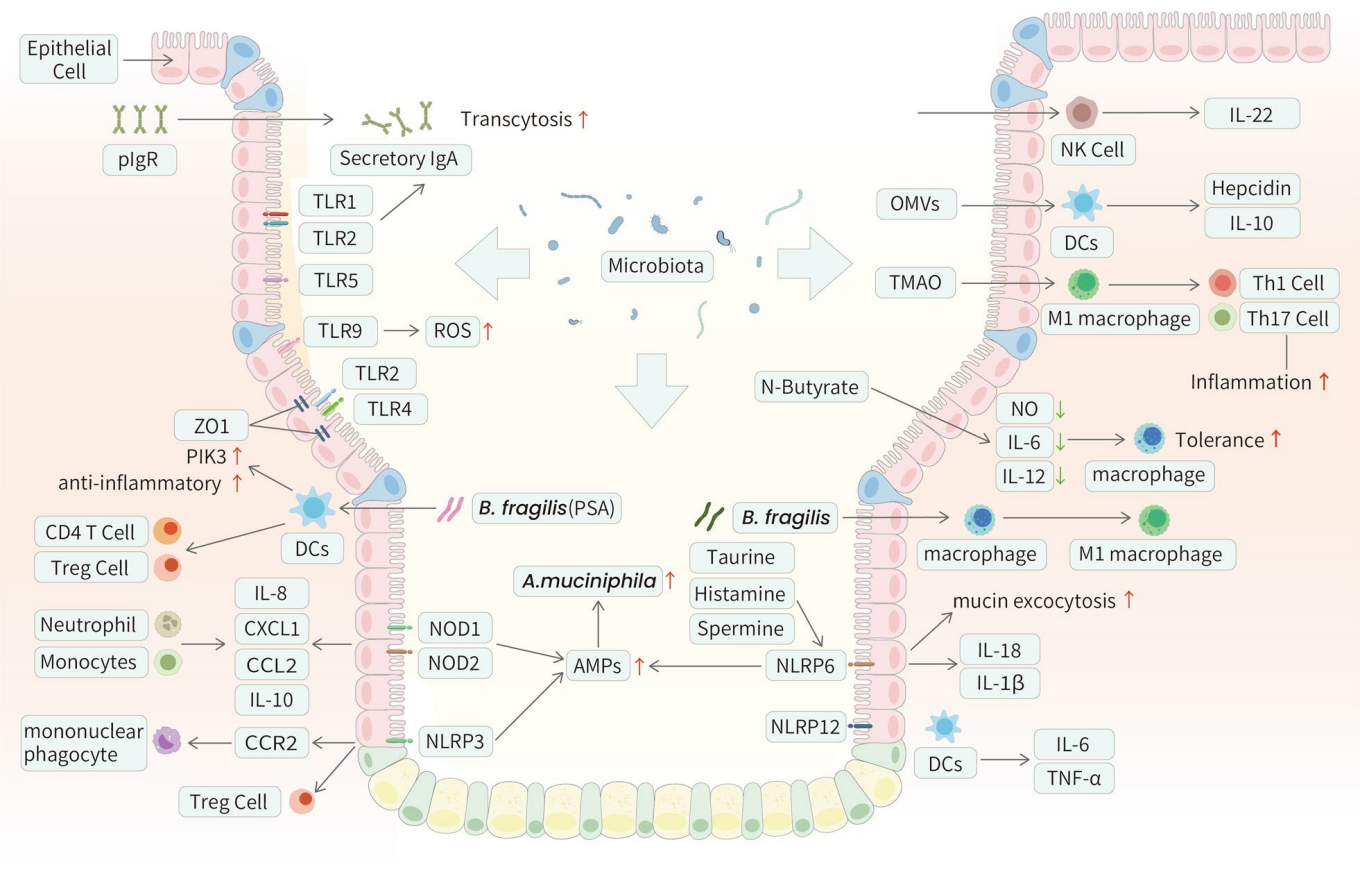
The interplay between the innate immune system and the gut microbiota in GI tract. Some mechanisms utilized by the gut microbiota to interact with the host innate immune system in GI tract are described above. The interplay between the gut and its microbiota is complex. The secretion of AMPs could be affected by *A.muciniphila*. PRRs are strongly affected by the presence of the gut microbiota. Microbiota-derived TLR and NOD ligands act directly on intestinal immunocytes and can activate inflammatory genes. *Bacteroides fragilis* stimulates the downstream PI3K pathway and activates the transcription of anti-inflammatory genes by co-operating TLR1/TLR2 heterodimer and Dectin-1. NLRs function to activate inflammatory caspases and cytokines to compost optimal microbiota and maintain intestinal homeostasis. Microbial metabolites taurine, histamine, and spermine have been identified to regulate the activation of NLRP6 inflammasome as well as the induction of downstream epithelial IL-18 and AMPs secretion. Innate immune cells, including macrophages, DCs, and NK cells, interact heavily with the gut microbiota. OMVs derived from *Bacteroides* elicit IL-10 production by DCs, as well as enhance the phagocytic functions of macrophages triggered by the bacteria themselves. The expression of the transcription factor RORγt and IL-22 of intestinal NK cells is conditioned by the commensal microbiota

AMPs are secreted by epithelial cells in the gut, mostly Paneth cells [43]. They are a crucial component of immunoreactive substances, and affect the innate immune system. As the first-line defender, AMPs modulate the immune system in response to a wide range of invasive pathogens. The most abundant AMPs, human defensin(HD) HD-5 and HD-6, modulate the microbiota in vivo via an increase in the abundance of *Akkermansia* sp [44]. In mouse models, the lack of pore-forming Orai1 was associated with high mortality due to severe intestinal bacterial dysbiosis, and the absence of AMP secretion from acinar cells was considered the major cause [45] (Fig. 2).

### Pattern recognition receptors (PRRs)

PRRs identify host receptors that recognize specific pathogen-associated molecular patterns (PAMPs), making PRRs a critical factor in defense against infectious pathogens [46]. Following activation by PAMPs, PRR signaling pathways produce AMPs, cytokines, chemokines, and apoptotic factors. These factors are expressed not only in innate immunity but also in various nonprofessional immune cells, such as intestinal epithelial cells (IECs) in the GI tract. The most well-studied PRRs are toll-like receptors (TLRs) and nucleotide oligomerization domain (NOD)-like receptors (NLRs) [47]. Understanding how microbes influence PRR-associated immune responses is fundamental for understanding gut microbiome homeostasis.

TLRs are widely expressed in the GI tract but differ significantly between the intestine and colon [48]. We focused on TLR4, TLR5, TLR9, and TLR2, which are involved in microbe recognition. In the context of the GI tract, TLR2 is expressed in mononuclear cells of the lamina propria, goblet cells, and enterocytes. TLR4 and TLR9 are expressed mainly in IECs [49]. In addition, TLR5 is expressed on the basolateral side of IECs in the colon, while its expression is restricted to Paneth cells in the small intestine [50]. TLRs are strongly affected by the presence of microbes [51]. In particular, we will discuss how TLR signaling mediates the crosstalk between microorganisms and IECs and how this structural and functional interplay primes immune cell responses in the gut mucosa. Microbial metabolites strongly regulate IEC proliferation, apoptosis, and differentiation [52]. These processes can be induced by the development of goblet cells that are activated by TLR2 and TLR4 [53]. The motility of intestinal smooth muscle could be another factor that impacts the differentiation of IECs, which is mediated by TLR4, TLR5, and TLR9 [54, 55]. Researchers have revealed that TLR2 stimulation effectively preserves tight junction-associated barrier integrity by promoting phosphoinositide 3-kinase (PI3K)/Akt-mediated cell survival via myeloid differentiation primary response gene 88 (MyD88) as well as the translocation of zona occludens 1 (ZO1) and occluding proteins [56]. Moreover, activation of TLR4 induces a loss of barrier function through the expression of myosin light chain kinase (MLCK) [57]. In addition, AMP and IgA transcytosis are highly dependent on TLR-mediated recognition of the gut microbiota [58, 59]. IECs control microbial invasion of the mucosa through the release of ROS into the lumen after TLR activation [60]. These results indicate that TLRs are involved in intercellular junctions, and that enhancing or disrupting intestinal epithelial barrier integrity depends on microbes. A typical example for understanding TLR–microbe interplay is the symbiont molecule polysaccharide A (PSA) of *Bacteroides fragilis (B.fragilis)*. PSA interacts with the TLR1/TLR2 heterodimer on DCs in cooperation with Dectin-1 to stimulate the downstream PI3K pathway, followed by the transcription of anti-inflammatory genes. This PSA-dependent immunomodulation is essential for presenting CD4 + T cells and Treg cells, which are critical for producing interleukin-10 (IL-10), which is the primary anti-inflammatory outcome [61, 62].

NLRs activate inflammatory caspases and cytokines and modulate inflammatory signaling pathways [63]. NOD1/NOD2 recognizes peptidoglycan in bacterial cells and activates the NF-κB/extracellular-signal-regulated kinase(ERK) /mitogen-activated protein kinase(MAPK) signaling pathway to mediate cytokine, chemokine, and antimicrobial peptide expression, thereby promoting the host immune response [64–66]. Specifically, stimulation of epithelial cells with NOD1 stimulatory molecules can induce the production of CXCL1, CCL2, IL-8, and AMPs, which are essential for recruiting neutrophils [67]. In NOD2(-/-) mice, inflammatory pathologies associated with the expansion of *Bacteroides vulgatus* were observed [68]. Researchers confirmed that NOD2 mediates CCL2-CCR2-dependent recruitment of inflammatory monocytes and promotes their production of IL-10 [69]. Moreover, the anti-inflammatory effects of *Lactobacillus salivarius Ls33* were abrogated in NOD2(-/-) mice [70]. NOD-like receptor thermal protein domain associated protein(NLRP)3, plays a well-defined role in intestinal homeostasis and protection against inflammation [71]. According to Seo et al. [72], *Proteus mirabilis* (*P. mirabilis*) can induce robust IL-1β release by meditating the recruitment of CCR2 mononuclear phagocytes. Similarly, Yao et al. [73] confirmed that the hyperactive NLRP3 inflammasome could remodel the gut microbiota by inducing IL-1β production. Furthermore, they observed enhanced production of AMPs and compensatory changes in local Treg cell levels to neutralize inflammation. Another well-studied inflammasome-forming NLR is NLRP6. Elinav et al. [74] described the novel regulatory mechanism of the NLRP6 inflammasome in which a deficiency of NLRP6 resulted in reduced IL-18 and IL-1β levels. Additionally, NLRP6 knockout mice had an increased abundance of *Akkermansia muciniphila (A.muciniphila)* [75]. Wlodarska et al. [76] further explored the regulatory effect of the NLRP6 inflammasome on the biogeographical distribution of the gut microbiota, and the authors suggested that NLRP6 mediates mucin granule exocytosis and subsequent mucous layer formation. In another study, Levy et al. [77] reported that taurine, histamine, and spermine activated NLRP6 inflammasome and induced downstream epithelial IL-18 and AMP secretion. In addition to inflammasome formation, NLRP12 suppresses NF-κB signaling and the expression of downstream inflammatory cytokines [78–81]. Two recent studies have connected NLRP12 with the gut microbiota in the contexts of colon inflammation and obesity. Chen et al. [82] found that microbial dysbiosis contributed to colitis in NLRP12 knockout mice. These mice exhibited increased expression of inflammatory cytokines, including tumor necrosis factor-α(TNF-α) and IL-6, by DCs, which was reversed by the administration of *Lachnospiraceae*. In addition, inflammation associated with obesity in NLRP12-deficient mice was attributed to the maintenance of beneficial microbiota [83] (Fig. 2).

### Macrophages

Macrophages are known as the first-line of defense against pathogens, but they also interact heavily with commensal bacteria [84]. *B. fragilis* enhances the phagocytic functions of macrophages by polarizing them to an M1 phenotype [85]. Researchers have shown that the gut microbiota promotes the interaction between IL-1β–secreting macrophages and colony-stimulating factor 2 (Csf2)-producing RORγt + innate lymphoid cells 3 (ILC3s) [86]. Several studies have explored the influence of microbial products on macrophages. By inhibiting the release of NO, IL-6, and IL-12, n-butyrate may assist in the tolerance of colon macrophages to commensals [87]. Furthermore, butyrate-enhanced antimicrobial activity was shown to be related to alterations in macrophage metabolism and increased LC3-associated antimicrobial clearance [88]. TMAO-polarized inflammatory macrophages induce a potent Th1 and Th17 response by modulating the microenvironment, which exacerbates inflammation-related diseases [89] (Fig. 2).

### Dendritic cells (DCs)

DCs are the most potent and versatile professional antigen-presenting cells (APCs), that can initiate the adaptive immune response and support innate immunity [90]. DCs can be divided into plasmacytoid DCs (pDCs) and conventional DCs (cDCs) [91, 92]. Researchers have suggested that cDCs cannot be fully activated due to insufficient interferon-I(IFN‐I) signaling. In other words, the gut microbiota, which is the major regulator of IFN-I secreted by pDCs, controls the basal state of DCs [93]. Another example of this crosstalk is the outer membrane vesicles (OMVs) derived from *Bacteroides thetaiotaomicron*. These OMVs are instrumental in eliciting regulatory IL-10 production by DCs [94]. In addition, Bessman et al. [95] reported that hepcidin produced by cDCs in response to microbiota-derived signals promoted intestinal homeostasis. (Fig. 2).

### Natural killer (NK) cells

NK cells are an important component of the innate immune system and account for up to 15% of all lymphocytes [96]. Researchers have suggested that the innate mucosal defense provided by a subset of intestinal NK cells is conditioned by the commensal microbiota, which expresses the transcription factors RORγt and IL-22 [97]. Four trials applying synbiotics or probiotics have shown that administration improved the gut microbiota composition and increased NK cell activity and the levels of associated cytokines [98–101]. More specifically, Qiu et al. [102] reported that the probiotic *Lactobacillus plantarum* can efficiently increase the expression of IL-22 mRNA and protein in NK cells, thereby mitigating intestinal epithelial barrier damage. (Fig. 2).

### B cells

**Fig. 3:**
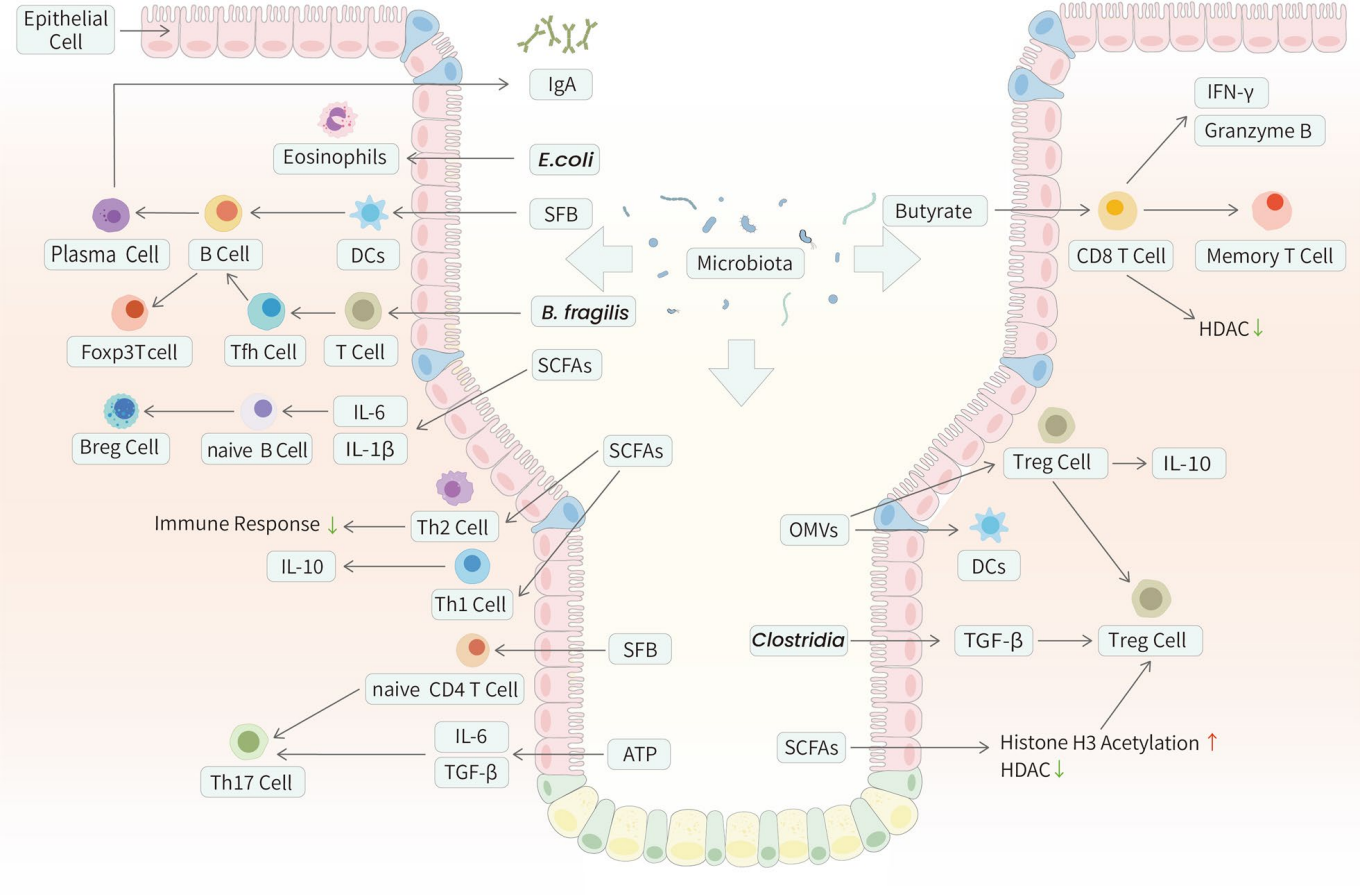
The interplay between the adaptive immune system and the gut microbiota in GI tract. Some mechanisms utilized by the gut microbiota to interact with the host innate immune system in GI tract are described above. Foxp3 + Treg cells promote maturation of B cells and production of secretary IgA. These contribute to the regulation of homeostatic microbiota composition and the maintenance of a non-inflammatory host-microbial relationship. CD8 + T cells can be activated by the intestinal microbiota and its metabolites. Butyrate, for instance, showed a direct antagonistic influence on the HDAC of CTLs and Tc17 cells, thereby promoting the expression of IFN-γ and granzyme B. As for Th cells, the adhesion of SFB to IECs is a common outcome of inducing homeostatic intestinal Th17 cells. Tfh cells, being another modulation target of gut microbiota modification, are essential for the production of plasma cells and memory B cells. The SCFAs have been demonstrated to regulate the size and function of the Treg cell pool

B cells are crucial mediators of intestinal homeostasis. By secreting immunoglobulins and cytokines, they assist in maintaining a noninflammatory host-microbe relationship [103, 104]. GF mice show a reduced amount of immunoglobulin A, a differentiated form of B-cell, and impaired B-cell responses [105]. The intestinal colonization of *E. coli*, *bifidobacteria*, and *segmented filamentous bacteria (SFB)* might promote B-cell maturation and enhance the specific IgA antibody response [106, 107]. This IgA response helps maintain gut microbiota homeostasis, thereby facilitating the expansion of Foxp3 + T cells and maturation of the gut immune system through a symbiotic regulatory loop [108]. The regulation of B cells by the gut microbiota and its products could be influenced by IgA, immune cells, chemokines, cytokines, or even B cells themselves [109]. More specifically, B-cell activating factors can be induced by IECs, DCs, T cells, and eosinophils. Together, these immune cells and cytokines can promote the differentiation and survival of IgA plasma cells [110–114]. Additionally, microbial metabolites such as SCFAs activate B-cell receptors (BCRs), inhibit histone deacetylases (HDACs), and increase adenosine triphosphate (ATP) levels [115, 116]. The differentiation of naïve B cells into regulatory B cells (Bregs) can be induced by intestinal microbiota-driven production of IL-1β and IL-6 [117] (Fig. 3).

### CD8 + T cells

T cells coordinate the immune response and directly kill damaged cells. These functions are mediated by CD4 + and CD8 + T cells, respectively. CD8 + T cells play central roles in controlling infections and cancer. These cells are known to secret IFN-γ and the protease granzyme B, which act synergistically to kill infected or tumorigenic cells [118]. CD8 + T cells can be activated by the intestinal microbiota and its metabolites, such as cytotoxic T lymphocytes (CTLs), to exert direct cytotoxicity and interact with other immune cells, especially in the tumor microenvironment (TME) [119]. Conversely, microbial dysbiosis exacerbates chronic inflammation and tumor susceptibility, thereby attenuating the activity of CD8 + T cells and sometimes even causing their exhaustion [120–123]. Moreover, butyrate had a direct antagonistic influence on the HDACs of CTLs and cytotoxic T lymphocyte 17 (Tc17) cells, thereby promoting the expression of IFN-γ and granzyme B [124]. Butyrate could also promote activated CD8 + T cell differentiation into memory cells [125]. Immunotherapy targeting the close interaction between CD8 + T cells and the gut microbiota is promising and will be discussed below (Fig. 3).

### Helper T (Th) cells

Th cells, which are differentiated from naïve CD4 + T cells, can orchestrate humoral and cellular immunity by facilitating the activation of immunocytes in a cytokine-dependent manner [126, 127]. Different subsets of Th cells show distinct functions in protective immunity and reactivity to the gut microbiota because of differences in the production of signature cytokines [128]. Th1 cells produce IFN-γ, IL-2, and TNF-α, and the expression of IL-4, IL-5, and IL-13 defines Th2 cells. Th17 cells are abundant within the GI tract and help regulate gut microbes. The signature cytokines of this cell subset include IL-17A, IL-17F, and IL-22 [129]. Th1 and Th2 cells exhibit functions that are regulated by the gut microbe-derived metabolites [130]. SCFAs are associated with an impaired ability to initiate a Th2 cell immune response [131]. Additionally, SCFAs can promote microbe antigen-specific IL-10 production in Th1 cells through GPR43 and induce the expansion of the Th1 transcription factor T-bet [132]. Furthermore, cancer patients display decreased plasma tryptophan(Trp) levels correlated with an increase in Th1-type immune activation markers [133]. The potential association between Th17 cells and gut microbes has been shown in different diseases. Specific alterations in the intestinal mucosa-associated microbiota were correlated with an increased number of intestinal Th17 cells and a high disease burden [134]. Preclinical models further verified this correlation by showing that augmenting the population of pathogenic colonic Th17 cells could promote tumorigenesis [135]. However, their causal relationships have not been proven. We propose that the delicate balance of plasticity makes Th17 cells potential pathogenic drivers of intestinal immune diseases [136–141]. Studies have shown that the gut microbiota and metabolites activate Th17 cells. The impaired plasticity of Th17 cells in the absence of the gut microbiota can be restored by microbial metabolites [142–144]. *SFB* is a representative example of a molecule that can induce homeostatic intestinal Th17 cells [145, 146]. Atarashi et al. [147] further demonstrated that the adhesion of *SFB* to IECs is a critical factor for inducing Th17 cells and antigen binding to pro-Th17 DCs. Another study revealed that Bifidobacterium adolescentis could influence Th17 cells in a similar manner [148–150]. Researchers have shown that ATP derived from commensal bacteria can activate a unique subset of lamina propria cells, namely, CD70high/CD11clow cells, which induce IL-6 and transforming growth factor(TGF)-β, leading to the differentiation of Th17 cells [151]. Moreover, different gut microbe-derived BA and SCFA metabolites regulate and modulate Th17 cell immunological function and differentiation [152, 153]. Various diets have also been shown to have complicated impacts on Th17 cells [154, 155] (Fig. 3).

### Follicular helper T (Tfh) cells

Another critical subset of Th cells is Tfh cells. In addition to assisting B cells in producing antibodies, Tfh cells are essential for germinal center (GC) formation, affinity maturation, and the production of memory B cells [156]. The maturation of Tfh cells is restricted in GF mice, resulting in diminished IgA development and disruptions in microbial homeostasis [111]. Alterations in the gut microbiota can be observed in Tfh cells when ATP-gated ionotropic P2X7 receptors are absent [157, 158]. Moreover, bacteria of the genus *Anaeroplasma* can increase intestinal IgA levels by inducing TGF-β in Tfh cells [159] *SFB* can induce the differentiation of Tfh cells and egress into systemic sites, thereby facilitating systemic Tfh cell responses and autoantibody secretion that can worsen diseases [160]. Microbiota-derived eATP can also regulate Tfh cell abundance [161]. Thus, the gut microbiota can be a modulatory target of Tfh cells to further impact intestinal immunity [162] (Fig. 3).

Some Treg cells are also found in B-cell follicles and were identified as T follicular regulatory (Tfr) cells. These cells can migrate into the GC, thereby inhibiting B-cell maturation and antibody production [163] *SFB,* which induces Tfh cells to promote autoimmune arthritis, has also exhibited the potential to influence systemic Tfr cells [164]. In addition, butyrate is an environmental cue that can induce the differentiation of Tfr cells, which can also ameliorate autoimmune arthritis [165].

### Regulatory T (Treg) cells

Treg cells, which differentiate from naïve CD4 + T cells, are an irreplaceable constituent of immunity and are involved in the maintenance of immunological self-tolerance and homeostasis. Treg cells express the transcription factor Foxp3 in the nucleus and CD25 and CTLA-4 on the cell surface [166]. These factors are modulated by gut microbial signals [167–170]. TGF-β, the physiological inducer of the transcription factor Foxp3 (associated with the development of Treg cells), can be induced by *Clostridia* [171, 172] *B. fragilis* has been shown to form OMVs, packed with capsular PSA, and increase IL-10 expression in Treg cells, and activate TLR2 ligation on T cells and DCs [173, 174]. SCFAs have been demonstrated to regulate the size and function of the Treg cell pool [175, 176]. Specifically, butyrate promotes histone H3 acetylation at the Foxp3 locus, and propionate inhibits HDACs [177, 178].

In summary, microbes exert positive and negative effects on the immune system of the GI tract, thus indicating their dual role in cancer progression. Gut microbiome homeostasis enhances the host immune response. However, dysbiosis and depletion of the gut microbiome interfere with the immune system abnormally by manipulating various innate and adaptive immune system components, which may further increase susceptibility to tumorigenesis. (e.g., inducing a loss of intestinal barrier function through the PRR signaling pathway; affecting B-cell differentiation and response; attenuating CD8 + T cells, even causing their exhaustion; causing impaired plasticity in Th17 cells; and restricting the maturation of Tfh cells). Specifically, different strains of gut microbes play different roles in regulating GI tract immunity. In the GI tract, *A.muciniphila*, *B.fragilis*, *Ls33*, *Lachnospiraceae*, *E. coli*, *bifidobacterial*, *SFB*, and *Bifidobacterium adolescentis* are associated with immune cell activation processes and exhibit anti-inflammatory properties. Moreover, strains like *Bacteroides vulgatus* displayed inflammatory pathologies, which might be involved in cancer progression. Microbial metabolites showed similar dual characteristics. Butyrate attenuates the inflammatory response, while TMAO promotes it.

## The gut microbiota and the efficacy of cancer immunotherapy

**Fig. 4:**
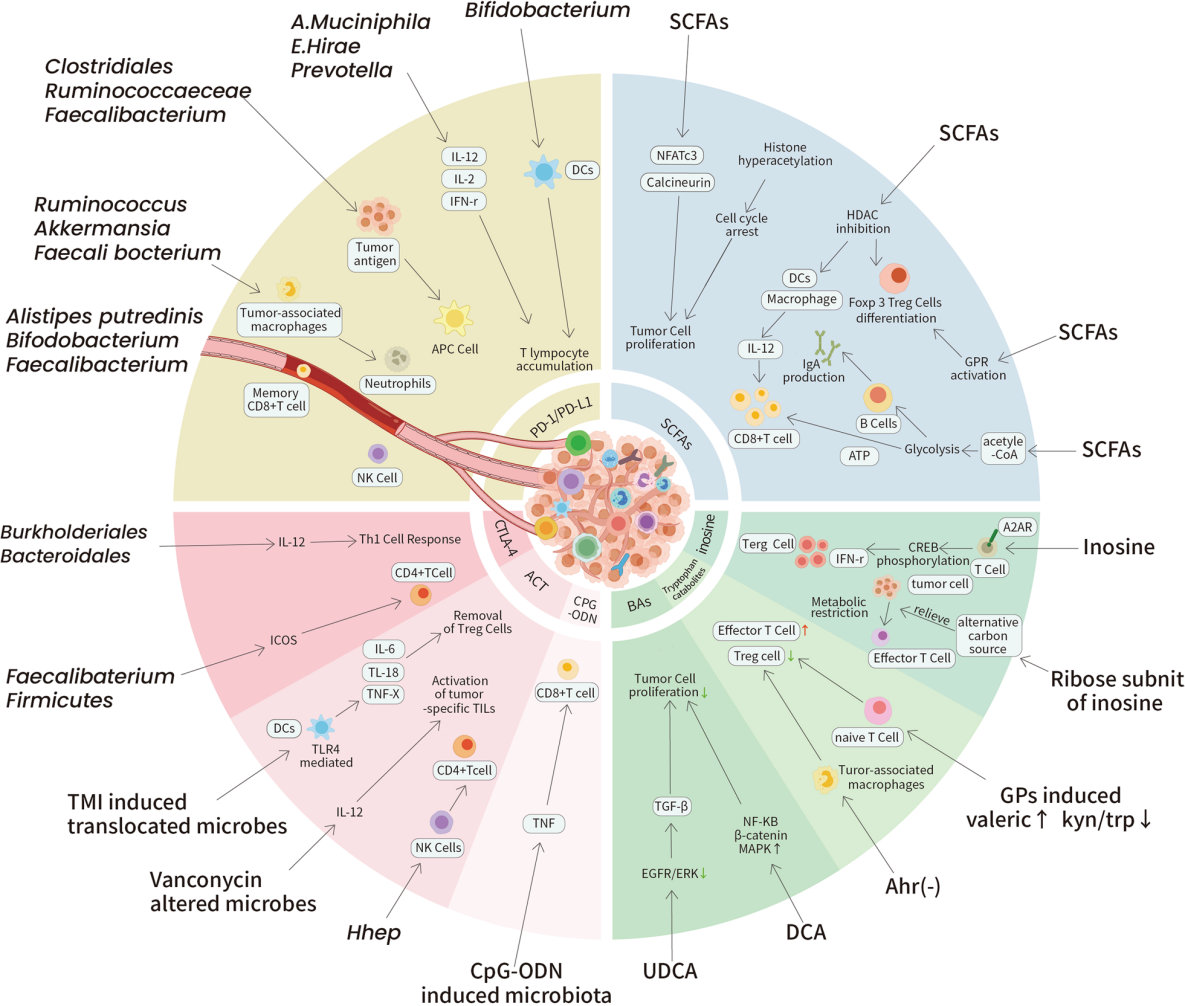
Selected mechanisms of how the gut microbiota impact cancer immunotherapies. Current studies have revealed the close link between the gut microbiota and the efficacy of cancer immunotherapy. Grouped by immunotherapies and metabolites, outlined here are some selected mechanisms utilized by the gut microbiota and its metabolites to regulate immunocyte activation, cytokine secretion, metabolism restriction and tumor cell proliferation inside the TME to influence cancer immunotherapy effects

The idea of cancer immunotherapy has evolved rapidly in the past few decades. Many types of immunotherapy have been developed to revive the immune system by suppressing the immunoinhibitory pathways commonly employed by tumor cells to escape immunosurveillance. A close link between the gut microbiota and cancer immunotherapy has slowly been unveiled with an increasing number of innovative studies. We outline the recent evidence in this field by type of immunotherapy (Additional file 1: Table S1) (Fig. 4).

### Antibodies against PD-1/PD-L1

PD-1 is a coinhibitory transmembrane receptor expressed on tumor-infiltrating lymphocytes (TILs) [179]. Within the TME, PD-1 binds to PD-L1 and consequently inhibits CTL-mediated cytolysis, as well as Fas-induced cellular apoptosis, thus allowing tumor cells proliferate indefinitely [180, 181]. Inhibitors of PD-1/PD-L1, such as nivolumab, pembrolizumab, and atezolizumabor, promote immune responses against cancer cells in clinical trials [182–187].

Moreover, landmark experiments have confirmed the association between antibodies against PD-1/PD-L1 and the gut microbiota. These preclinical trials have explored the hallmark mechanisms of this crosstalk: (1) alterations in the gut microbiota composition caused by immune checkpoint inhibitors(ICIs), (2) the effects of gut microbes on intestinal immune cells, (3) induced metabolic changes affecting the immune response of commensals, and (4) the accumulation of immunocytes in the TME caused by the gut microbiota. Specifically, this crosstalk was first explored by Sivan et al. [22]. Their data suggested that *Bifidobacterium* could augment DC functions and enhance CD8 + T-cell priming and accumulation in the TME. Routy et al. [188] confirmed the correlation between the abundances of different microbes (*A.muciniphila and E.hirae*) and PD-1/PD-L1 blockade efficacy. Mechanistically, these researchers demonstrated that the antitumor effect was restored in an IL-12-dependent manner by increasing the recruitment of CCR9 + CXCR3 + CD4 + T lymphocytes into the TME. Another study indicated that *Prevotella* and *A.muciniphila* improved the therapeutic efficacy of PD-1/PD-L1 inhibitors and *Bacteroides* led to poorer efficacy. Researchers have speculated that changes in the gut microbiota affect glycerophospholipid metabolism, thereby altering the expression of IFN-γ and IL-2 in the TME [189]. In mice with breast cancer (BC), anti-PD-1 therapy increased the abundance of *Bifidobacterium*, *Lactobacillus*, and *Adlercreutzia* [190].

Analogous clinical studies were implemented in the following years, and the results validated the correlation between the gut microbiota composition and the therapeutic efficacy of ICIs in clinical trials beyond preclinical models.

In trials involving metastatic melanoma (MM) patients, contradictory results showed that no single species could be regarded as an entirely consistent predictive factor. In terms of mechanism, Gopalakrishnan et al. [191] reported increased abundances of *Clostridiales*, *Ruminococcaceae*, and *Faecalibacterium* in responders(R) and suggested that increasing antigen presentation and improving effector T-cell function in the TME could enhance antitumor immune responses. Matson et al. [192] performed FMT to transfer R-enriched bacteria into colonized mice and observed an increased frequency of DCs and augmented T-cell responses. Other studies have shown that specific bacterial species are associated with R and nonresponders(NRs) [193, 194] and that carriers of specific bacterial taxa exhibit a better cancer prognosis [195, 196].

Multiple studies on the systemic immune responses of cancer patients ranging from those with melanoma to those with non-small cell lung carcinoma (NSCLC) have detected a greater frequency of memory CD8 + T cells and NK cells in the periphery of R enriched with *Alistipes putredinis Bifidobacterium longum*, and *Prevotella copri* [197]. A group in the United States found that mice model with transplanted gut microbes had improved ICI efficacy when the TME was enriched with immunocytes [198]. Other studies have also demonstrated a diverse array of molecular features in the gut microbiota during immunotherapy modulation [199–206]. Taken together, the findings are conflicting; thus, continued research efforts are needed to establish causal relationship between different microbes and ICI treatment efficacy. Similarly, studies focusing on other rare thoracic malignancies are needed, although initial data have been provided [207].

Not until 2019 did studies start focusing on predicting responses to PD-1/PD-L1 immunotherapy based on the gut microbiota composition in the context of hepatocellular carcinoma (HCC). Zheng et al. [208] reported that the dynamic nature of commensals plays an important role in ameliorating oxidative stress injury and host inflammatory responses in antitumor therapy. Another study revealed that the antitumor functions of certain bacterial species could be a result of SCFA production and bile acid metabolism [209]. Although multiple studies have demonstrated that better ICI efficacy in HCC patients appears to be correlated with a favorable gut microbiota [210–212], one recent study failed to confirm such a positive association in patients with HCC [213].

Compared with those of the solid tumors mentioned above, little is known about the direct impact of individual intestinal nonpathogenic bacteria on the therapeutic outcomes of ICIs in renal cell carcinoma (RCC). Derosa et al. [214] observed a positive association between *D. formicigenerans* and CD8 + CD69 + T cells as well as negative associations between *C. clostridioforme* and CD137/4.1BB expressing CD4 + T lymphocytes and memory CXCR5-CCR6-CCR4-CCR10-CXCR3 + CD8 + T cells. Salgia et al. [215] also identified several species that were presumably correlated with therapeutic benefits.

Although a significant amount of research has been dedicated to revealing how the gut microbiota influences the carcinogenesis of colorectal carcinoma (CRC), little is known about the regulatory mechanisms involved in the efficacy of ICIs. In a recent study, *F. nucleatum* was connected to the activation of the stimulator of interferon genes (STING) signaling pathway as well as the accumulation of IFN-γ + CD8 + TILs [216]. To better understand how individual bacterial species modulate ICI therapy, future studies are needed to better characterize any shared functionalities among different microbial communities.

The negative impact of *H. pylori* on immunomodulation raises the concern that *H. pylori* infection may suppress immune responses to cancer immunotherapy [217, 218]. Researchers have confirmed that *H. pylori* infection decreases the effectiveness of cancer immunotherapies by inhibiting DCs and suppressing CD8 + T-cell responses [219].

### Antibodies against cytotoxic T lymphocyte-associated antigen 4 (CTLA-4)

CTLA-4 is a major negative receptor of T cells and has upregulated expression upon T-cell activation [220–226]. Inhibitors of CTLA-4, such as ipilimumab and tremelimumab, are thought to boost antitumor immunity due to the strong immunosuppressive effects of CTLA-4 [227–231]. Mechanistically, anti-CTLA-4 blockade affects the Th1 subset of CD4 T cells that express an inducible costimulator (ICOS) [232, 233]. Additionally, both effector T cells and Tregs are the primary targets of anti-CTLA-4 mediated blockade [234, 235].

Studies have revealed the mechanisms by which different species of gut microbiota improve the clinical outcomes of anti-CTLA-4 immunotherapy. Initially, an altered gut microbiota was thought to activate IL-12-dependent Th1 immune responses, thereby facilitating antitumor effects [236, 237]. Chaput et al. [238] confirmed that prolonged progression-free survival (PFS) and overall survival (OS) in patients enriched with *Firmicutes* was mediated by increased ICOS induction levels of CD4 + T cells and sCD25 levels. A recent study suggested that the antitumor efficacy of CTLA-4 blockade is negatively correlated with the proportion of the microbial metabolite butyrate since systemic butyrate is capable of inhibiting ipilimumab-mediated DC maturation and the CD28 signaling pathway (Additional file 1: Table S1) [239].

### Adoptive cell transfer (ACT)

While ICI efficacy relies on the presence of tumor-reactive T cells [240], ACT may be a good strategy for treating poorly immunogenic types of cancer [241]. There are two approaches to ACT: (1) isolating TILs from the TME and (2) genetically modifying blood-derived T cells to express chimeric antigen receptor (CAR). Both approaches require in vitro T-cell manipulation before reinfusion into patients [242–247]. Considering the obstacles to the application of ACT, interventions modulating the immune microenvironment, such as gut microbiota modifications, have become a central issue [248, 249].

Paulos et al. [250] reported for the first time that translocated microbes could augment the function of ACT therapy by triggering the TLR4 pathway. Activating this pathway stimulates DCs and increases the secretion of proinflammatory cytokines in the gut. Similarly, other studies also revealed enhanced ACT efficacy after vancomycin supplementation, which induced IL-12 expression to increase the number and activity of tumor-specific TILs [251]. Adoptive transfer of naïve *Helicobacter hepaticus* (*Hhep*)-specific CD4 + T cells has been shown to contribute to antitumor immunity in CRC. Mechanistically, researchers have discovered that increased *Hhep* levels stimulate tertiary lymphoid structures (TLSs), which further activate NK cells and CD4 + T cells [252]. Recently, Smith et al. [253] demonstrated a close correlation between a high abundance of *Ruminococcus*, *Bacteroides*, and *Faecalibacterium* and better responses to CD19 CAR T-cell therapy in patients. Collectively, these findings, although preliminary, have not revealed the exact mechanisms by which bacterial taxa and metabolites influence ACT immunotherapy outcomes, especially CAR-T-cell therapy outcomes (Additional file 1: Table S1) [254].

### Unmethylated cytidine phosphate guanosine oligonucleotide (CpG-ODN) therapy

CpG-ODNs possess immunostimulatory effects and potential antitumor activity [255]. They interact with TLR9 in B cells and plasmacytoid DCs to initiate a signaling cascade that activates the NF-κB pathway and various cell types and induces the production of cytokines and chemokines [256]. Thus, CpG-ODN injections were initially promoted for their immunotherapeutic potential, and recent studies have focused on applying CpG-ODNs as an adjuvant to other cancer treatments [257–259].

Iida et al. [119] identified several species associated with CpG-ODN efficacy. These associations suggest that the gut microbiota affects immunotherapy by inducing TNF production and manipulating tumor-associated myeloid cells. These findings confirmed that commensals affect the outcomes of patients receiving CpG-ODN therapy by regulating inflammatory responses in the TME (Additional file 1: Table S1).

## Microbial metabolites and the efficacy of cancer immunotherapy

Metabolites derived from the gut microbiota have been identified as important regulators of the development and function of immune cells [17, 260, 261]. Given their complicated interactions with the immune system, multiple studies have focused on how they impact local and systemic antitumor immune responses, especially in the context of ICI therapy (Fig. 4). These heavily studied metabolites can be divided into three subgroups according to their origin and synthesis: (1) metabolites produced by the gut microbiota from dietary components, (2) metabolites produced by the host and modified by the gut microbiota, and (3) metabolites synthesized de novo by the gut microbiota. We will discuss the latest evidence about the potential mechanisms underlying these interactions for each of these groups.

### Metabolites produced by the gut microbiota from dietary components

#### SCFAs

In the intestine, dietary fiber can be fermented into SCFAs by the gut microbiota [262]. These SCFAs act as signaling molecules that regulate host physiology and immune processes, specifically by inhibiting HDACs or activating G protein-coupled receptors (GPRs) [87, 263–266]. Multiple studies have confirmed the association between gut microbiota-derived SCFAs and the long-term benefits of ICI treatment in cancer [202, 267–269]. However, Coutzac et al. [239] identified the antagonist effect of SCFAs that limits anti-CTLA-4 activity. Here, we will discuss the critical role that SCFAs play in the immune system, which demonstrates their antitumor effects in cancer immunotherapy.

SCFAs directly inhibit the proliferation of tumor cells. Researchers have shown that butyrate can inhibit tumor cell proliferation by decreasing the activation of nuclear factor of activated T-cell (NFAT)c3 and calcineurin [267]. Additionally, propionate produced by *A. muciniphila* promotes tumor cell apoptosis [268] In addition, SCFAs can induce histone hyperacetylation by inhibiting HDACs, leading to cell cycle arrest [269].

Moreover, SCFAs activate immune cells to augment antitumor immune responses. SCFAs can modulate intestinal macrophages and DCs through the inhibition of HDACs [87, 265, 270, 271]. Research has also shown that SCFAs modulate the suppressive function and differentiation of Foxp3 + Treg cells in an HDAC-dependent manner to establish immunological homeostasis in the gut [175, 177, 178, 272]. Singh et al. [273] showed that the GPR-butyrate interaction is another signaling factor that is involved in the differentiation of Treg cells. SCFAs also improved the efficacy of anticancer therapy by influencing cytotoxic CD8 + T cells. The antitumor effect was boosted by the inhibition of class I HDAC enzymes via an IL-12-dependent signaling pathway [274, 275]. The metabolic promotion of glycolysis and oxidative phosphorylation in CD8 + T cells induced by SCFAs provides energy for immune cells [276]. In addition, SCFAs increase acetyl-CoA levels to modulate energy metabolism in B cells to support antibody production [112].

There are also contradictory findings showing restricted antitumor activity of anti-CTLA-4 in the face of high systemic levels of butyrate [239], leading to poor clinical response to treatment with ICIs. Although the mechanism through which SCFAs affect the efficacy of ICIs remains ambiguous, the SCFA-associated immunomodulatory pathway and its relevant clinical trials are still a promising area of research.

#### Tryptophan catabolites

Tryptophan catabolites, which mostly result from the degradation of dietary proteins, are critical contributors to intestinal and systemic homeostasis [277]. These proteins act as ligands for the aryl hydrocarbon receptor (AhR) [278], which is a ligand-inducible transcription factor in host cells that assists in immune responses [279, 280]. Accumulating evidence has confirmed the antitumor effect of targeting these microbial metabolites in cancer treatment.

Clinical research has shown that a decreased ratio of serum kynurenine(Kyn)/ Trp improves ICI treatment efficacy [281, 282]. In concert, studies have further demonstrated that T-cell proliferation can be inhibited by high Kyn/Trp ratios, which consequently worsens patient prognosis [283]. Another clinical trial revealed the immunosuppressive activity of 3-hydroxyanthranilic acid (3-HAA), which is a downstream metabolite in the kynurenine pathway [284].

High levels of AhR expression have been recognized as a signal for rapid disease progression. Hezaveh et al. [285] observed the activation of AhR in tumor-associated macrophages (TAMs) by microbiota-derived tryptophan metabolites in pancreatic ductal adenocarcinoma (PDAC). Moreover, deletion of AhR reduced tumor growth, increased the number of IFNg + CD8 + T-cells, and improved the efficacy of ICI treatment.

Indole-3-carboxaldehyde (3-IAld) exhibits great potential in modulating the immune response at the interface between microbes and the host immune system [286]. Researchers have found that 3-IAld in alters the composition of the gut microbiota and induces SCFAs production [287]. In addition, 3-IAld has been shown to alleviate irAEs by activating the AhR/IL-22 pathway, which targets the epithelial barrier to help maintain mucosal homeostasis [288].

According to Huang et al. [289], interventions such as prebiotics assist in the accumulation of the tryptophan catabolite valeric acid. Decreased Kyn/Trp ratios could suppress Treg cells and activate effector T cells, which will eventually enhance the efficacy of anti-PD-1 immunotherapy. In summary, these findings support the oncogenic effect of the kynurenine pathway and the antitumor effect of indoles.

### Metabolites produced by the host and modified by the gut microbiota

#### Bile acids

Bile acids (BAs) are a group of metabolites synthesized from cholesterol and then formed by the gut microbiota [290]. Limited knowledge is available regarding the correlation between ICI treatment outcomes and BAs, while relatively more is known about the mechanism through which BAs modulate the host immune system.

A recent study revealed distinct BA features in Rs and NRs to ICI-treated HCC. Specifically, ursodeoxycholic acid (UDCA) was significantly more abundant in Rs, whereas lithocholic acid (LCA) was more abundant in NRs [291]. The antitumor effect of UDCA has been widely reported [292]. Various signaling pathways, immune cells, and cytokines, such as the epidermal growth factor receptor (EGFR)/ERK signaling pathway, NKT cells, and TGF-β, are involved in the protective effect of UDCA [293–295].

Secondary BAs such as deoxycholic acid (DCA) activate EGFR and protein kinase C, thus causing DNA damage and apoptosis and eventually leading to cancer cell proliferation [296–299].

### Metabolites synthesized de novo by the gut microbiota

#### Inosine

A recent study identified that *A. muciniphila* and *B. pseudolongum* utilize the inosine-adenosine 2A receptor(A2AR) signaling pathway to improve the efficacy of ICI therapy. The authors presumed that inosine activates T cells and reprograms the TME [300]. Based on their findings and other relevant studies, we identified several potential mechanisms through which inosine may influence immune responses to ICI therapy.

The immunomodulatory effects of inosine on immune cells could be a critical factor. Activation of the inosine-A2AR-cAMP-PKA signaling pathway leads to phosphorylation of the transcription factor cAMP response element–binding protein (CREB) [300]. Other research has shown that the microbiota–inosine–A2AR axis can influence the differentiation and expansion of Treg, CD8 + T, Th1, and Th2 cells and the production of cytokines [301–305].

Furthermore, inosine can support cell growth and T-cell functions as an alternative metabolic substrate. The high metabolic demands of cancer cells can limit the capacity of effector T cells by restricting available nutrients [306–308]. Wang et al. [309] demonstrated that inosine can relieve tumor-imposed metabolic restrictions on T cells. Specifically, effector T cells utilize the ribose subunit of inosine to activate central metabolic pathways and generate ATP and biosynthetic precursors.

#### Peptidoglycan

In a recent study, NOD2-active muropeptides generated by active enterococci with orthologs of the NlpC/p60 peptidoglycan hydrolase SagA were shown to improve the efficacy of ICI immunotherapy [310]. Further mechanistic exploration revealed that microbiota-derived peptidoglycans augment CD8 + T cells that express granzyme B and a particular monocyte population characterized by Cx3cr1 and Nr4a1 expression [39]. Accordingly, researchers suggested that specialized peptidoglycan remodeling activity and muropeptide-based strategies could be regarded as the future of next-generation immunotherapy.

#### Immune-related adverse events and the gut microbiota

A large spectrum of autoimmune responses is associated with ICIs due to their impact on immune cell activation [311]. Inflammatory side effects termed immune-related adverse events (irAEs) are frequently linked to the gastrointestinal tract, endocrine glands, skin, and liver during ICI treatment [312–316]. These potential irAEs reveal the necessity of multidisciplinary, collaborative management across the clinical spectrum [317, 318]. In addition to identifying microbial signatures associated with the efficacy of ICI therapy, the microbiota composition and dysbiosis in the gut have also shown a connection with the incidence of irAEs (Additional file 1: Table S2).

In terms of immunotherapy-related colitis, multiple studies have identified various microbial signatures and related signaling pathways that mediate the proinflammatory side effects of ICIs. Dubin et al. [319] reported a correlation between the abundance of specific bacterial taxa and subsequent colitis development. This report was followed by several studies that identified more irAE-colitis-associated gut microbes ranging from *Firmicutes* families to *Streptococcus spp* [196, 200, 209, 236, 238]. In addition to studies on colitis-induced bacteria, other studies have suggested that *Bifidobacterium* ameliorates colitis [320]. Researchers have demonstrated that *Bifidobacterium breve* and *Lactobacillus rhamnosum* can enhance the suppressive function of Treg cells by stimulating an IL-10/IL10Ra signaling loop [321].

These discoveries have provided opportunities to target gut microbes using strategies such as FMT or probiotics to decrease intestinal toxicity. Researchers in a case series utilizing FMT to abrogate ICI-associated colitis observed an increase in the proportion of Treg cells within the colonic mucosa [322]. Additionally, administration of the probiotic *L. reuteri* could ameliorate the immunopathology associated with ICIs by affecting the local number of ILC3s [323]. The microbial metabolite 3-IAld has demonstrated therapeutic potential in maintaining epithelial barrier function in the gut, which could help alleviate ICI-induced intestinal toxicity [286].

With the increased use of ICIs, irAEs are no longer limited to colitis but include all kinds of related diseases, such as diarrhea, pancreatitis, pruritus, and thyroid dysfunction. Researchers have identified various characteristics of the gut microbiome related to the increasing risk of irAEs [324–326]. Usyk et al. [327] applied this widely studied connection to predict the incidence of irAEs.

In summary, utilizing the microbiota composition as a prediction tool and therapeutic target for irAEs in ICI-treated patients may be a promising direction for treatment.

## Gut microbiota modifications in response to cancer immunotherapy

**Fig. 5:**
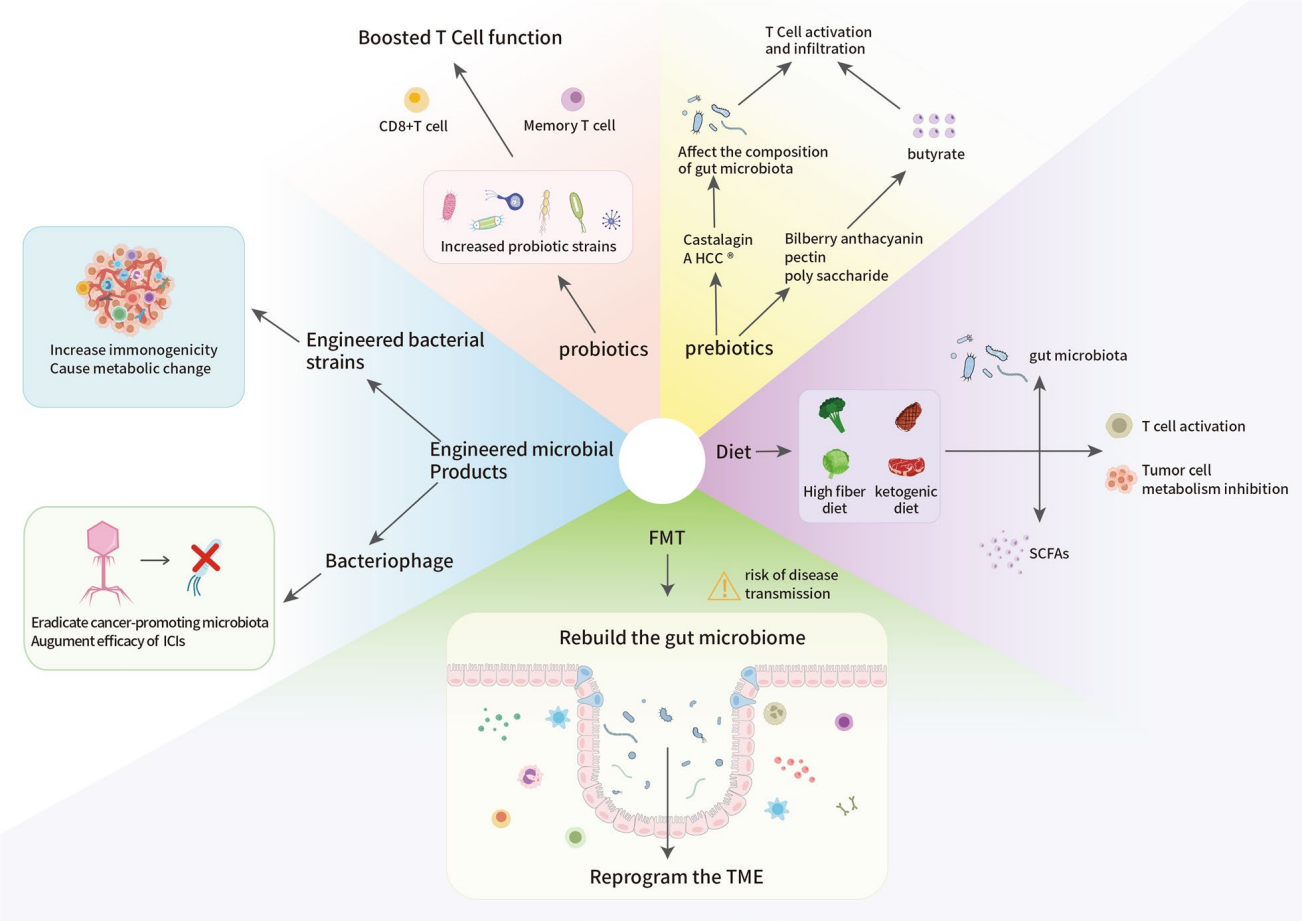
Future intervention strategies to modificate gut microbiota in cancer immunotherapy. Targeting the association between the gut microbiome and cancer immunotherapy, modifying the gut microbiota with the latest intervention technologies could significantly advance the quality of individualized treatment. Listed here are the potential mechanisms behind the five microbiota modification strategies, which could be used to promote the efficacy of cancer immunotherapy in a precise manner. These intervention strategies are developed mainly based on current views of the crosstalk between the gut microbiota and the immune system. FMT, dietary regulation, probiotics, prebiotics, and engineered microbial products all can alter intestinal bacteria to enhance anti-tumor immune responses inside the TME, which consequently improve the efficacy of cancer immunotherapy

Accumulating evidence has revealed how the gut microbiota and its metabolites interact with the host immune system to regulate antitumor immunity and immunotherapy responses. Therefore, modifications of the gut microbiota to enhance ICI treatment efficacy are promising approaches for therapeutic development. Here, we review preclinical and clinical trials that aimed to improve the clinical outcomes of patients treated with ICIs by altering gut microbes (Fig. 5). The main methods used for this purpose include FMT, dietary regulation, probiotics, prebiotics, and engineered microbial products.

### FMT

FMT is a well-established clinical approach for modulation of the gut microbiota [328]. Transplantation of the gut microbiota from a healthy donor restores intestinal microbial diversity in the recipient [329]. Currently, FMT is recommended by the FDA for treating recurrent *Clostridium difficile* infection [330].

Considering the unique microbial features of ICI responders, it is tempting to presume that FMT is applicable in immunotherapy. Several preliminary trials have explored coupling FMT with immunotherapy, and their results have indicated that FMT could induce the differential expression of T-cell and NK cell-related pathways in ways that control tumor growth and ameliorate the immune response [188, 191, 192, 331].

Three recent studies have investigated the feasibility of introducing FMT through oral stool capsules in patients treated with ICIs. All of these studies revealed desirable outcomes, including an increased abundance of bacteria associated with response to anti-PD-1 therapy, activation of CD8 + T cells, and a decreased amount of IL-8-expressing myeloid cells. The microbiota sources were obtained from healthy stool donors [23, 261, 332]. These observations confirmed that FMT could alter the microbiota composition and reprogram immune and inflammatory factors to increase the efficacy of ICIs [333]. The safety data from Routy et al. [332] confirmed that FMT combined with anti-PD-1 therapy did not increase the incidence of irAEs. Additionally, Spreafico et al. utilized a microbial consortium, Microbial Ecosystem Therapeutic 4 (MET4), as an alternative to FMT in combination with ICIs in patients with advanced solid tumors. Their results suggested no worsening of ICI-associated irAEs when using MET4 [334]. Given these promising results, there are many ongoing clinical trials investigating the exact mechanism behind FMT-induced enhancement of ICI efficacy in larger patient cohorts (Additional file 1: Table S3).

Recently, two live microbiome therapeutic products were approved by the FDA: RBX2660 and SER-109. Clinical trials on these products have shown that they reduce the incidence of recurrent *Clostridioides difficile* infection (rCDI) with a low risk of adverse events related to treatment. We summarized the detailed trial design and results of these products(Table 3).

**Table 3:** The latest FDA approved live microbiome therapeutic products

Microbiome therapeutic product name	Participants	Trial design	Group	Results			
				Treatment success rates (after 8 weeks) (%)	Sustained clinical response (between 8 weeks to 6 months)	Adverse events	The number of engrafting dose species (after 8 weeks)
RBX2660	267 participants with rCDI were included, 180 in RBX2660 group and 87 in Placebo group	1. A randomized, double-blind trial	RBX2660	70.6	92.1%	55.6% (after 6 months)	–
		2. Intention-to-treat population used in statistical analysis	Placebo	57.5	90.6%	44.8% (after 6 months)	–
SER-109	182 participants with rCDI were included, 89 in SER-109 group and 93 in Placebo group	1. A randomized, double-blind trial	SER-109	87.6	–	51.1% (after 8 weeks)	66
		2. Intention-to-treat population used in statistical analysis	Placebo	60.2	–	52.2% (after 8 weeks)	56

Based on their innovativeness, RBX2660 and SER-109 were granted Breakthrough Therapy Status, Fast Track, and Orphan Drug designations by the FDA [335, 336].

However, there is also considerable risk during FMT [337]. For example, a whole transplantation of the gut microbiota may sabotage the existing boundary of beneficial bacteria in the recipient, thereby causing infectious diseases [338]. Therefore, professional guidelines should be put in place to mandate presurgical safety screenings for donors, define standardized duration and delivery methods for the procedure, and build machine learning models that can to predict responses to minimize FMT-associated risks [339–341].

### Dietary regulation

Recent studies have revealed the potential regulatory effect of diet on the gut microbiota [342]. Multiple studies have proven that dietary interventions can alter the composition of the gut microbiome. For instance, the standard Western diet (which is high in fat and carbohydrates and low in fiber) could induce gut dysbiosis, as it causes an increase in *Firmicutes*, *Proteobacteria*, *Mollicutes*, *Bacteroides spp.*, *Alistipes spp.*, *Bilophila spp.*, *Enterobacteriaceae*, *Escherichia*, *Klebsiella*, and *Shigella* while decreasing the abundance of beneficial bacteria *Bacteroidetes*, *Prevotella*, *Lactobacillus spp.*, *Roseburia spp.*, *E. rectale*, *Bacillus bifidus* and *Enterococcus*, leading to increased BA secretion and decreased downstream SCFA production [343–345]. Moreover, low-fat, high-fiber diets can improve the gut microbiome composition by shifting the microbiota composition toward and increase in the beneficial bacteria *Prevotella* and *Bacteroides* and a decreased in *Firmicutes* [346]. Therefore, dietary regulation via the gut microbiota could be a promising clinical strategy to improve the efficacy of cancer treatment [347–352].

One clinical study that focused on the impact of the food-gut axis on the response to ICIs revealed a positive correlation between high-fiber diets and improved responsiveness to anticancer immunotherapy. Specifically, higher expression of genes related to T-cell activation and the interferon response were observed in the high-fiber diet group, which were likely induced by fiber-fermenting bacteria through the production of SCFAs [353].

A ketogenic diet, which is a high-fat, low-protein, and low-carbohydrate diet, is well known for its ability to inhibit lactate-mediated tumoral immunosuppression and tumor cell metabolism [354–356]. Ferrere et al. studied the efficacy of combining a ketone-rich diet with immunotherapy [357] and reported that supplementation with ketone bodies could re-establish therapeutic responses when ICI treatment failed to reduce tumor growth on its own. A ketogenic diet could induce changes in the gut microbiota composition, leading to the expansion of CXCR3 + T cells and inhibition of the IFNγ-mediated upregulation of PD-L1 expression on myeloid cells.

Currently, many tentative clinical trials aimed at characterizing diet-induced alterations in the gut microbiota and their possible effects on immunotherapy efficacy are underway to better understand their relationship (Additional file 1: Table S3).

### Probiotics

Probiotics are defined as “live microorganisms which, when administered in adequate amounts, confer a health benefit to the host” [358]. Probiotics have been applied to prevent and treat multiple diseases [355–357] and specifically for cancer, Lactobacillus spp. and Bifidobacterium spp. strains were capable of relieving dysbiosis, enhancing anticancer immunity, and improving ICI treatment efficacy in recent studies [359–362].

The utilization of single probiotic strains has yielded exciting therapeutic effects when combined with cancer immunotherapy. *Bifidobacterium* supplementation has been shown to play a key role in improving ICI efficacy [22, 363]. The probiotics *Clostridium butyricum* and *Lactobacillus rhamnosus*, and antibiotic-resistant lactic acid bacteria may also improve the therapeutic efficacy of ICIs as they increase the number of beneficial bacteria and reshape functional metagenomes [24, 364–366]. In terms of *A. muciniphila*, researchers have identified an IL-12-dependent mechanism by which *A. muciniphila* triggers the recruitment of CCR9 + CXCR3 + CD4 + T lymphocytes into the TME to increase the efficacy of ICI treatments [188]. Increased T-cell function was also observed in CTLA-4 mAb-treated patients administered *L.acidophilus*. Zhuo et al. [367] reported that ICI efficacy could be enhanced by increasing the abundance of CD8 + T cells and effector memory T cells, as well as by decreasing the abundance of Treg cells and M2 macrophages in the TME.

Compared to single probiotic strains, a bacterial consortium may better represent the collective properties of the gut microbiota. Tanoue et al. [368] applied a bacterial consortium containing 11 commensal strains in tumor-bearing mice and identified a mechanism or enhancing ICI efficacy that was dependent on CD103 + DCs and major histocompatibility class Ia cells. A recent study validated the use of probiotics as a stand-alone therapy for treating tumors, where a mix of four *Clostridiales* species could exert antitumor effects by activating CD8 + T cells and increasing the immunogenicity of tumors [369].

Nevertheless, there is conflicting evidence on the benefits of probiotics marketed as dietary supplements [370]. Suez et al. [371] identified a delayed reconstitution of the gut mucosal microbiota using an 11-strain probiotic cocktail. Inconsistent clinical results also exist of the agonist effects of probiotic strains and formulations in immunotherapy have also been reported [353]. More efforts are needed to gain a thorough understanding of the effects of probiotics on immune responses and cancer immunotherapy (Additional file 1: Table S3).

### Prebiotics

A prebiotic is defined as a substrate that is selectively utilized by host microorganisms to confer a health benefit [372]. Studies have shown that prebiotics can assist in promoting immunomodulatory effects, as well as stimulating the gut barrier and enhancing metabolic functions [373].

Prebiotics may improve the immunomodulatory effects of ICIs by altering the adundance of SCFAs. Researchers have shown that natural prebiotics, such as bilberry anthocyanin, pectin, the plant polysaccharide inulin, and ginseng polysaccharides, modulate anti-PD-1 therapy. These prebiotics can increase the amount of beneficial SCFAs, which further induces systemic memory T-cell responses and increases T-cell infiltration and activation in the TME [289, 374–377]. Alternatively, artificial prebiotics such as AHCC® (a standardized extract of cultured Lentinula edodes mycelia) and castalagin also enhanced ICI efficacy by altering the gut microbiota composition and enhancing T-cell functions within the TME [378, 379].

### Engineered microbial products

With the development of genetic technology, engineered microbial products have attracted research interest worldwide. In contrast to the innate microbiota, these engineered microbes are designed to be sensitive to disease signals and respond to them at the site of onset [380]. They also contain bacteriophages, which modulate the composition of the gut microbiota.

To date, multiple reports have demonstrated the reliable delivery of antitumor benefits by engineered bacterial strains in many different contexts [381–385]. Here, we discuss how these microbes could be applied as a complement to anticancer immunotherapy. Binder et al. [386] demonstrated a powerful new therapeutic approach, that combines *Salmonella typhimurium* with PD-L1 blockade to activate the expansion of tumor-specific CD8 + T cells, resulting in the eradication of tumors. Similarly, Mkrtichyan et al. [387] observed an increase in CD8 + T-cell infiltration and antigen-specific immune responses in the periphery during anti-PD-1 immunotherapy after the administration of *Listeria monocytogenes*. These studies supported the hypothesis that microbes could indeed establish a more immunogenic microenvironment. Another approach to improve antitumor effects would be to enable metabolic modulation. Intertumoral injection of the Nissle 1917 *E.coli* strain increased the intracellular L-arginine concentration, triggered T-cell infiltration, and amplified the efficacy of PD-L1 blockade [388]. However, further technical refinements are still needed before the full-fledged clinical application of engineered bacteria can be achieved [389].

The utilization of bacteriophages as microbe-targeting vectors to induce immunomodulation has attracted extensive research interest [290, 390]. Bacteriophages promote the eradication of cancer-promoting commensals while maintaining their influence on the surrounding microbiota. A bacteriophage-guided, biotic–abiotic hybrid nanosystem could also provide precise phage release within the TME to accurately remove only pro-tumoral bacteria. For instance, *F. nucleatum*-specific phages have been shown to augment the efficacy of ICIs as well as first-line chemotherapy treatments [391, 392]. Notably, studies have revealed that correlations between specific bacteriophages and bacteria appear to be associated with FMT outcomes [393, 394].

These engineered microbial products are promising for immunotherapy development, and more studies are needed to explore their potential application.

## Challenges and future perspectives

In this review, we systematically examined current studies on the intricate relationship between the gut microbiota and the host immune system. Given the dynamic interactions among the gut microbiota, its metabolites, and various cancer immunotherapies including ICI, ACT, and CpG-ODN therapy, future studies should focus on discovering the underlying mechanisms of this modulatory effect, in addition to investigating distinct microbiota compositions. Recently, there has been accumulating evidence that the gut microbiota is a leading cause of irAEs in cancer immunotherapy. To minimize irAEs and improve immunotherapy safety, more studies are needed to develop novel interventions targeting commensal bacteria. Additionally, after reviewing the current therapeutic trials utilizing FMT, diet control, probiotics, prebiotics, and engineered microbial products combined with immunotherapy, we believe that there is still a tremendous need to explore the design of personalized methods of microbiota modification and strategies to optimize therapeutic efficacy.

Recent research on microbiota-cancer immunotherapy interactions shares the common concern of heterogeneity in trial design [5], which can be attributed to the lack of a uniform methodology during sample allocation, technology utilization, data quality control, and data analysis. To address this issue, a consortium-level effort is needed to construct a standardized protocol specifying certain requirements for microbial specimen type and origin, sample handling environment, and microbiota bioinformatics analysis [395]. In addition to the study design, dynamic alterations in the gut microbiota and time-dependent disease progression could also induce heterogeneity [396, 397]. Therefore, consistent monitoring of the microbial composition throughout the disease course or exploration of the predictable patterns of microbial communities needs to be incorporated as a part of study protocols [398]. A recent study developed a computational method that exhibited promising potential for monitoring the dynamic alterations in gut microbes. This approach revealed the associations between drug exposure and the microbiome at high resolution, indicating the capacity to predict microbial changes and patient outcomes [399].

Moreover, the high degrees of biological inter- and intrapersonal variability of the gut microbiota imply that there is much more to learn in terms of individual heterogeneity [400]. Emerging spatial multiomics tools, especially single-cell techniques, are invaluable in deciphering the heterogeneous configurations of individuals at the bacterial strain level [401, 402]. Despite the accumulating evidence of improved therapeutic outcomes in humans and preclinical model mice, there are still gaps in our knowledge regarding the modulating effects of the gut microbiota that hindering its clinical application. Most importantly, most studies have focused solely on observing the correlation between the gut microbiota and treatment outcomes rather than exploring the existence of any causality. Because the gut microbiota functions as a whole, the impact of modifying individual bacterial strains may have different effects on the collective properties of the entire gut microbiota beyond an individual strain. To advance the current research from association-based to mechanism-based, the application of synthetic biology in the human microbiota might be a critical tool [403, 404].

In terms of gut microbiota modification, more functional studies and prospective clinical trials are needed to translate preclinical interventions targeting the gut microbiota into clinical applications in humans. One main challenge of applying experimental interventions in the clinic is that humans and animals do not share the same immune system. Another factor that cannot be ignored is differences in the gut microbiome composition and richness between rodents and humans. These limitations have restricted the translation of preclinical studies focusing on the gut microbiota. Therefore, the construction and characterization of the human gut microbiota in vitro could significantly improve the quality of individualized immunotherapy [405]. Furthermore, in situ genome engineering of the microbiota has also demonstrated promising potential for the regulation of existing microbial communities, which suggests its future utilization in the manipulation of cancer immunotherapy outcomes [406].

In summary, our knowledge about the intricate relationships among the gut microbiota, the host immune system, and cancer immunotherapy are still limited. By combining artificial intelligence applications with the emerging advances we mentioned above [407], future research should provide further insights into the crosstalk between the microbiota and clinical outcomes of immunotherapies, thus paving the way for the clinical application of gut microbiota interventions, as well as the development of personalized medicine for cancer management.

### Supplementary Information

**Additional file 1.** Additional files of studies and trials related to the gut microbiome.

## Data Availability

Not applicable.

## Abbreviations

SCFAs: Short-chain fatty acids
TMAO: Trimethylamine N-oxide
GF: Germ-free
PD-1/PD-L1: Programmed cell death protein-1/programmed cell death protein-1-ligand 1
FMT: Fecal microbiota transplantation
GI: Gastrointestinal
CTX: Cyclophosphamide
Th cell: Helper T cell
Treg cell: Regulatory T cell
AMPs: Antimicrobial peptides
HD: Human defensing
PRRs: Pattern recognition receptors
PAMPs: Pathogen-associated molecular patterns
IECs: Intestinal epithelial cells
TLRs: Toll-like receptors
NOD: Nucleotide oligomerization domain
NLRs: Nucleotide-binding domain and leucine-rich repeat-containing receptors
PI3K: Phosphoinositide 3-kinase
MyD88: Myeloid differentiation primary response gene 88
ZO1: Zona occludens 1
MLCK: Myosin light chain kinase
PSA: Polysaccharide A
*B. fragilis*: *Bacteroides fragili* *s*
IL-10: Interleukin-10
ERK: Extracellular-signal-regulated kinases
MAPK: Mitogen-activated protein kinases
NLRP: NOD-like receptor thermal protein domain associated protein
*P. mirabilis*: *Proteus mirabilis*
*A. muciniphila*: *Akkermansia muciniphila*
TNF-α: Tumor necrosis factor-α
DCs: Dendritic cells
Csf2: Colony-stimulating factor 2
ILC3: Innate lymphoid cells 3
DCs: Dendritic cells
APCs: Antigen-presenting cells
pDCs: Plasmacytoid dendritic cells
cDCs: Conventional dendritic cells
IFN‐I: Interferon-I
OMVs: Outer membrane vesicles
NK cell: Natural killer cell
*SFB*: *Segmented filamentous bacteria*
BCR: B cell receptors
HDAC: Histone deacetylase
ATP: Adenosine triphosphate
Bregs: Regulatory B cells
CTLs: Cytotoxic T lymphocytes
TME: Tumor microenvironment
Tc17: Cytotoxic T lymphocyte17
Trp: Tryptophan
TGF: Transforming growth factor
Tfh cell: Follicular helper T cell
GC: Germinal center
Tfr cells: T follicular regulatory cells
ICIs: Immune checkpoint inhibitors
TILs: Tumor-infiltrating lymphocytes
BC: Breast cancer
MM: Metastatic melanoma
R: Responders
NR: Non-responders
NSCLC: Non-small-cell lung carcinoma
HCC: Hepatocellular carcinoma
RCC: Renal cell carcinoma
CRC: Colorectal carcinoma
STING: Stimulator of interferon genes
CTLA-4: Cytotoxic T lymphocyte-associated antigen-4
ICOS: Inducible costimulatory
PFS: Progression-free survival
OS: Overall survival
ACT: Adoptive cell transfer
CAR: Chimeric antigen receptor
*Hhep*: *Helicobacter hepaticus*
TLSs: Tertiary lymphoid structures
CpG-ODN: Unmethylated cytidine phosphate guanosine oligonucleotides
GPRs: G protein-coupled receptors
NFAT: Nuclear factor of activated T-cell
AhR: Aryl hydrocarbon receptor
Kyn: Kynurenine
3-HAA: 3-Hydroxyanthranilic acid
TAM: Tumor-associated macrophage
PDAC: Pancreatic ductal adenocarcinoma
3-IAld: Indole-3-carboxaldehyde
BAs: Bile acids
UDCA: Ursodeoxycholic acid
LCA: Lithocholic acid
EGFR: Epidermal growth factor receptors
DCA: Deoxycholic acid
A2AR: Adenosine 2A receptor
CREB: CAMP response element–binding protein
irAEs: Immune-related adverse events
rCDI: Recurrent clostridioides difficile infection
