# Supplementary material for: Critical role of the gut microbiota in immune responses and cancer immunotherapy
Source: J Hematol Oncol. 2024 May 14;17:33. doi: 10.1186/s13045-024-01541-w (PMC11094969; doi:10.1186/s13045-024-01541-w)
Supplement: Supplementary file 1 — Additional file 1. Additional files of studies and trials related to the gut microbiome. [file 13045_2024_1541_MOESM1_ESM.docx]

**Additional file**

**Supplemental Table 1. Studies describing the relationship between the gut microbiota and cancer immunotherapy**

| **Type of immunotherapy** | **Cohort** | **Associated bacterium** | **Major findings** | **Mechanistic exploration** | **Ref.** |
| --- | --- | --- | --- | --- | --- |
| Anti-PD-1/PD-L1 therapy | Mice bearing melanoma | *Bifidobacterium* | *Bifidobacterium* improved the efficacy of antitumor immunotherapy | Augmented DCs function;  Enhanced CD8+ T cell priming and accumulation in the TME | 22 |
| Anti-PD-1/PD-L1 therapy | Mice bearing melanoma;  Patients with various tumors | *A.muciniphila,*  *E.hirae,*  *Faecalibacterium* | *A.muciniphila, E.hirae* and *Faecalibacterium*: related to PD-1/PD-L1 blockade efficacy | Increased the recruitment of CCR9+CXCR3+CD4+T lymphocytes;  Restored the antitumor effect in an IL-12-dependent manner | 188 |
| Anti-PD-1/PD-L1 therapy | Mice bearing CRC | *Prevotella*,  *A.muciniphila,*  *Bacteroides* | *Prevotella* and *A.muciniphila*: related to the better therapeutic efficacy;  *Bacteroides*: related to the poor efficacy | Affected the metabolism of glycerophospholipids;  Altered the expression of IFN-γ and IL-2 in the TME | 189 |
| Anti-PD-1/PD-L1 therapy | Mice with BC | *Bifidobacterium,*  *A.muciniphila* | *Bifidobacterium, A.muciniphila*: elevated by PD-1 bolckade; | -- | 190 |
| Anti- PD-1/PD-L1 and CTLA-4/or alone treatment | Patients with melanoma | *Faecalibacterium prausnitzii, Holdemania filiformis, Bacteroides thetaiotamicron,*  *Dorea formicigenerans* | *Faecalibacterium prausnitzii, Holdemania filiformis, and Bacteroides thetaiotamicron*: enriched in PD-1 and CTLA-4 blockade R;  *Dorea formicigenerans*: enriched in PD-1 blockade R | -- | 193 |
| Anti-PD-1/PD-L1 therapy | Patients with melanoma | *Clostridiales, Ruminococcaceae, Faecalibacterium, Bacteroidales,*  *E.coli,*  *Anaerotruncus colihominis* | *Clostridiales, Ruminococcaceae, Faecalibacterium* enriched in R; *Bacteroidales, Anaerotruncus colihominis and E.coli* enriched in NR | Increased antigen processing and presentation;  Improved effector T cell function in the periphery and the TME;  Higher levels of Tregs and MDSCs with a blunted cytokine response | 191 |
| Anti-PD-1/PD-L1 therapy | Patients with melanoma | *Bifidobacterium longum,*  *Collinsella aerofaciens,*  *Enterococcus faecium* | *Bifidobacterium longum, Collinsella aerofaciens,* and *Enterococcus faecium* enriched in R | Increased frequency of DCs;  Augmented T cell responses;  Decreased Tregs in the periphery;  Reduced tumor control | 192 |
| Anti-PD-1/PD-L1 therapy | Patients with melanoma | *Streptococcus parasanguinis*,  *Bacteroides massiliensis*,  *Peptostreptococcacea* | *Streptococcus parasanguinis* and *Bacteroides massiliensis*: longer survival; *Peptostreptococcacea*: worse prognosis | -- | 195 |
| Anti-PD-1/PD-L1 therapy | Patients with melanoma | *Actinobacteria,*  *Lachnospiraceae,*  *Bacteroides,*  *Proteobacteria* | *Actinobacteria* and *Lachnospiraceae*: enriched in non-progressors of cancer;  *Bacteroides* and *Proteobacteria*: enriched in progressors of cancer | -- | 196 |
| Anti-PD-1/PD-L1 therapy | Patients with melanoma from 5 observational cohorts | *Bifidobacterium pseudocatenulatum*, *Roseburia* spp.*, A.muciniphila* | *Bifidobacterium pseudocatenulatum*, *Roseburia* spp.*,* and *A.muciniphila*: associated with R | -- | 194 |
| Anti-PD-1/PD-L1 therapy | Patients with NSCLC | *Alistipes putredinis,*  *Bifidobacterium longum,* *Prevotella copri,*  *Ruminococcus* | *Alistipes putredinis, Bifidobacterium longum*, and *Prevotella copri*: enriched in R; *Ruminococcus*: enriched in NR | Increased frequency of memory CD8+ T cell and NK cell in the periphery | 197 |
| Anti-PD-1/PD-L1 therapy | Patients with NSCLC | *Lactobacillus*, *Clostridium,* *Syntrophococcus, Bilophila*, *Sutterella*, and *Parabacteroides* | *Lactobacillus*, *Clostridium*, and *Syntrophococcus*: enriched in R; *Bilophila*, *Sutterella*, and *Parabacteroides* enriched in NR | Elicited T cell mobilization to the tumor | 199 |
| Anti-PD-1/PD-L1 therapy | Patients with NSCLC | *Ruminococcaceae UCG13*,  *Agathobacter* | *Ruminococcaceae UCG13* and *Agathobacter*: predicting beneficial responses to ICIs | Increased frequencies of effector CD4+ and CD8+ T cells in the circulation and CD8+ T-cell infiltration in the tumor | 200 |
| Anti-PD-1/PD-L1 therapy | Patients with NSCLC | *Parabacteroides,*  *Methanobacteriaceae,*  *Veillonella*,  *Selenomonadales*,  *Negativicutes* | *Parabacteroides* and *Methanobacteriaceae*: better prognosis of ICI treated cancer;  *Veillonella*, *Selenomonadales*, and *Negativicutes*: worse prognosis | -- | 201 |
| Anti-PD-1/PD-L1 therapy | Patients with NSCLC | *Granulicatella,*  *Ruminococcus bromii, Dialister* spp.  *Sutterella* spp. | *Granulicatella*: enriched in R;  *Ruminococcus bromii, Dialister and Sutterella spp*: enriched in NR | -- | 202 |
| Anti-PD-1/PD-L1 therapy | Patients with NSCLC | *Bifidobacterium bifidum* | *Bifidobacterium bifidum*: enriched in R | Potentiated IFN-γ production; Enhanced biosynthesis of immune-stimulating molecules and metabolites | 204 |
| Anti-PD-1/PD-L1 therapy | Patients with NSCLC | *Alistipes*, *Anaerostipes*,  *Desulfovibrio*,  *Actinomycetale,*  *Odoribacteraceae,*  *Rikenellaceae,*  *Faecalibacterium*,  *Bifidobacterium,*  *Fusobacterium* | *Alistipes*, *Anaerostipes*, *Desulfovibrio*, *Actinomycetale, Odoribacteraceae, Rikenellaceae,Faecalibacterium*, and *Bifidobacterium*: enriched in R;  *Fusobacterium*: enriched in NR | Increased density of CD8+T cell | 203 |
| Anti-PD-1/PD-L1 therapy | Patients with NSCLC | *A.muciniphila* | *A.muciniphila*: enriched in R | -- | 205 |
| Anti-PD-1/PD-L1 therapy | Patients with NSCLC | *Clostridia*,  *Bacteroidia,*  *A. muciniphila* | *Clostridia*, *Bacteroidia* and *A. muciniphila*: enriched in R | -- | 206 |
| Anti-PD-1/PD-L1 therapy | Mice with lung cancer;  Patients with NSCLC; | *Bacteroides,*  *Blautia,*  *A.muciniphila,*  *Faecalibacterium* | *Bacteroides, Blautia,* *A.muciniphila*, and *Faecalibacterium*: enriched in R | Enrichment in cytotoxic CD8+ IFNγ+ T cells CXCR3+ CD4+ T cells, neutrophils, and TAMs in the TME;  Increased type 1 immunity | 198 |
| Anti-CTLA4 and/or PD-1/PD-L1 treatment | Tumor bearing mice;  Patients with NSCLC | *H. pylori* | *H. pylori*-infected mice: less responsive to ICIs | Inhibited DC activation;  Interferring with the tumor-specific CD8+ T cell responses;  Decreasing the effectiveness of ICIs | 219 |
| Anti-PD-1/PD-L1 therapy | Patients with advanced thoracic carcinoma | *A.muciniphila,* *Enterococcaceae*, *Enterobacteriaceae*, *Carnobacteriaceae,* and *Clostridiales Family XI* | *A.muciniphila,* *Enterococcaceae*, *Enterobacteriaceae*, *Carnobacteriaceae,* and *Clostridiales Family XI*: enriched in R, prolonged PFS | Improved DC function and tumor-specific CD8+ T cell responses;  Induced the accumulation of IFN-γ-producing CD8 T cells | 207 |
| Anti-PD-1/PD-L1 therapy | Patients with BCLC | *Lactobacillus,*  *Bifidobacterium dentium*,  *Streptococcus thermophilus,*  *Ruminococcaceae* spp.  *A.muciniphil*  *E.coli,*  *Proteobacteria* | *Lactobacillus, Bifidobacterium dentium*, *Streptococcus thermophilus*, *Ruminococcaceae* spp. and *A.muciniphil*: enriched in R;  *E.coli* and *Proteobacteria*: enriched in NR | Ameliorating oxidative stress injury; Suppressing the host inflammatory response;  Prevented increases in intestinal permeability and systemic immunosuppression | 208 |
| Anti-PD-1/PD-L1 therapy | Patients with HCC | *Faecalibacterium*,  *Bacteroidales,*  *Clostridiales,*  *Ruminococcaceae* | *Faecalibacterium*: prolonged PFS; *Bacteroidales*: shortened PFS, enriched in NR;  *Clostridiales* and *Ruminococcaceae*: enriched in R | -- | 210 |
| Anti-PD-1/PD-L1 therapy | Patients with HCC | *Firmicutes*,  *Bacteroidetes,*  *Prevotella,*  *A.muciniphila* | Enriched *A.muciniphila* and higher ratio of *Prevotella* species to *Bacteroidetes*: R; a skewed *Firmicutes*/*Bacteroidetes* ratio (< 0.5 or > 1.5): NR | -- | 211 |
| Anti-CTLA4 and/or PD-1/PD-L1 treatment | Patients with HCC | *A.muciniphila,*  *Bifidobacterium,*  *Enterobacteriaceae* | *A.muciniphila* and *Bifidobacterium*: positively associated with disease control; *Enterobacteriaceae*: negatively associated with disease control | modulating the production and use of SCFAs | 212 |
| Anti-PD-1/PD-L1 therapy | Patients with HCC | *Bifidobacterium,*  *Acidaminococcus*,  *Coprococcus* | No positive association between the gut microbiome and the efficacy of ICIs;  *Bifidobacterium, Acidaminococcus*, and *Coprococcus* associated with disease control | -- | 213 |
| Anti-PD-1/PD-L1 therapy | Patients with HCC and BTC | *Lachnospiraceae*,  *Erysipelotrichaceae,*  *Ruminococcaceae,*  *Firmicutes*,  *Bacteroidetes,*  *Veillonellaceae* | *Lachnospiraceae*, *Erysipelotrichaceae,* *Ruminococcaceae*: enriched in R;  *Bacteroidetes*: enriched in R (BTC);  *Firmicutes*: enriched in R (HCC);  *Veillonellaceae*: enriched in NR | related to SCFAs production and bile acid metabolism | 209 |
| Anti-PD-1/PD-L1 therapy | Patients with RCC | *A. muciniphila,*  *B. salyersiae,*  *D. formicigenerans*  *C. clostridioforme* | *A. muciniphila* and *B. salyersiae*: enriched in R; *D. formicigenerans*: positively associated with CD8+CD69+ T cells;  *C. clostridioform*: negatively associated with CD137/4.1BB expressing CD4+ T lymphocytes and memory CXCR5-CCR6-CCR4-CCR10-CXCR3+CD8+ T cells | -- | 214 |
| Anti-PD-1/PD-L1 therapy | Patients with RCC | *A.muciniphila, Bifidobacterium adolescentis*,  *Barnesiella intestinihominis*,  *Odoribacter splanchnicus*, *Bacteroides eggerthii* | *A.muciniphila, Bifidobacterium adolescentis*, *Barnesiella intestinihominis*, *Odoribacter splanchnicus*, and *Bacteroides eggerthii*: related to clinical benefits | -- | 215 |
| Anti-PD-1/PD-L1 therapy | Patients with CRC | *F. nucleatum* | *F. nucleatum*: positively associated with the enhanced efficacy of ICIs | Activating STING signaling pathway; Increasing the accumulation of IFN-γ+ CD8+ TILs | 216 |
| Anti-CTLA-4 therapy | Patients with melanoma | *B.fragilis,*  *B.thetaiotaomicron,*  *Burkholderiales* | *B.fragilis, B.thetaiotaomicron and Burkholderiales*: increased in R | Activating the IL-12–dependent TH1 immune responses | 236 |
| Anti-CTLA-4 therapy | Patients with melanoma | *Faecalibacterium*,  *other Firmicutes* | *Faecalibacterium* and *other Firmicutes*: enriched in R | Inducing ICOS expression on CD4+ T cells; Increasing sCD25 expression;  Decreasing circulating α4+β7+ T cells and CD4+ Treg cells | 238 |
| Anti-CTLA-4 therapy | Patients with melanoma | *Faecalibacterium,*  *Gemminger*,  *Bacteroides* | *Faecalibacterium* and *Gemminger* : linked to long-term clinical benefit;  *Bacteroides*: linked to poor clinical outcome | SCFAs limit CTLA-4 blockade efficacy: restraining the maturation of DCs and the accumulation of tumor-specific T cells and memory T cells | 239 |
| ACT therapy | Tumor bearing mice | -- | Antibiotics or LPS signaling components use: reduced anticancer efficacy of ACT;  TBI: augmented function of ACT | Triggering the TLR4 pathway;  Activating DCs;  Increased secretion of pro-inflammatory across the gut | 250 |
| ACT therapy | Mice with HPV-associated cancers | *Bacteroides*,  *Parabacteroides* | *Bacteroides* and *Parabacteroides*: enriched in R | Raised systemic Cd8α+ DCs; induced TH1 immune responses; increased the abundance and activity of tumor-specific TILs | 251 |
| CAR-T therapy | Patients with B-cell malignancies | *Ruminococcus*,  *Bacteroide*s,  *Faecalibacterium* | *Ruminococcus*, *Bacteroide*s, and *Faecalibacterium*: enriched in R | -- | 253 |
| CpG-ODN therapy | Tumor bearing mice | *Ruminococcus,*  *Alistipes shahii*  *Lactobacillus* | *Ruminococcus* and *Alistipes shahii*: enriched in R;  *Lactobacillus*: enriched in NR | Inducing the production of inflammatory cytokine by tumor-associated innate myeloid cell;  Regulating the inflammatory response in the TME | 119 |

| **Supplemental Table 2. Studies describing the relationship between the gut microbiota and irAEs** | | | | | |
| --- | --- | --- | --- | --- | --- |
| **Type of irAEs** | **Type of immunotherapy** | **Associated bacterium** | **Major findings** | **Mechanistic exploration** | **Ref.** |
| Colitis | Anti-CTLA-4 therapy | *Bacteroidaceae,*  *Rikenellaceae,*  *Barnesiellaceae* | Increased abundance of *Bacteroidaceae*, *Rikenellaceae* and *Barnesiellaceae*: correlated with resistance to the development of colitis | Reduced capacity for microbe-mediated production of B vitamins and polyamine transport | 319 |
| Colitis | Anti-CTLA-4 therapy | *B. thetaiotaomicron,*  *B. fragilis* | Intestinal reconstitution of *B.thetaiotaomicron* and *B. fragilis*: amelioration of colitis | Affecting IL-12-dependent TH1 immune responses | 236 |
| Colitis | Anti-CTLA-4 therapy | *Firmicutes,*  *Bacteroidetes* | *Firmicutes:* colitis associated;  *Bacteroidetes:* no colitis-associated | A low proportion of peripheral blood regulatory T cells: colitis associated | 238 |
| Colitis (Diarrhea) | Anti-PD-1/PD-L1 therapy | *Prevotellamassilia timonensis* | enrichment of *Prevotellamassilia timonensis:* patients with more severe diarrhea | -- | 209 |
| Colitis | Anti-CTLA-4 therapy | *Bifidobacterium* | *Bifidobacterium*: amelioration of colitis | Modulating metabolic functions of Treg cells | 320 |
| Colitis | Anti-CTLA-4 therapy | *Bifidobacterium,*  *Lactobacillus* | *Bifidobacterium and Lactobacillus*:  amelioration of colitis | Enhancing both the mitochondrial fitness and the IL-10-mediated suppressive functions of intestinal Treg cells | 321 |
| -- | Anti-PD-1/PD-L1 therapy | *Lactobacillaceae,*  *Raoultella,*  *Akkermansia,*  *Agathobacter* | *Lactobacillaceae, Raoultella, and Akkermansia:* *associated with a less severe irAE profile*  *Agathobacter: associated with more severe irAE profile* | -- | 200 |
| -- | Anti-PD-1/PD-L1 therapy | *Streptococcus,*  *Lachnospiraceae* | *Streptococcus* and *Lachnospiraceae*:  associated with the insurgence of irAEs | -- | 196 |
| Diarrhea | Anti-PD-1/PD-L1 therapy | *Firmicutes,*  *Bacteroidetes* | *Bacteroidetes*: resistance to immune-related diarrhea;  *Firmicutes:* positively associated with diarrhea | -- | 324 |
| Immune‐related acute pancreatitis (irAP) | Anti-CTLA4 and/or PD-1/PD-L1 treatment | *Alistipes,*  *Bacteroides,*  *Lachnospiraceae,*  *Bacteroidetes*/*Firmicutes* ratio | *Low Bacteroidetes*/*Firmicutes* ratio and *Alistipes* and *Bacteroides, high Lachnospiraceae: baseline of irAP* | -- | 325 |
| -- | Anti-PD-1/PD-L1 therapy | *Streptococcus,*  *Paecalibacterium,*  *Stenotrophomonas,*  *Faecalibacterium,*  unidentified_*Lachnospiraceae* | Higher abundance of *Streptococcus*, *Paecalibacterium*, and *Stenotrophomonas*: severe irAEs;  higher abundance of *Faecalibacterium* and unidentified_*Lachnospiraceae*: mild irAEs | -- | 326 |
| -- | Anti-CTLA4 and/or PD-1/PD-L1 treatment | *Bacteroides dorei,*  *Bacteroides vulgatus* | *Bacteroides dorei*: high risk of irAEs;  *Bacteroides vulgatus*: low risk of irAEs | -- | 327 |

| **Supplemental Table 3. Selected clinical trials combine the gut microbiome modification and cancer immunotherapy** | | | | | | |
| --- | --- | --- | --- | --- | --- | --- |
| **Trial ID** | **Study title** | **Cancer type** | **Interventions** | **Study phase** | **Primary outcome measures** | **Location** |
| **FMT application in cancer immunotherapy** | | | | | | |
| NCT03353402 | Altering the Gut Microbiota of Melanoma Patients Who Failed Immunotherapy Using Fecal Microbiota Transplantation (FMT) From Responding Patients | Melanoma | FMT | Phase 1 | 1.Incidence of FMT-related Adverse Events;  2.Proper implant engraftment | Israel |
| NCT05286294 | MITRIC: Microbiota Transplant to Cancer Patients Who Have Failed Immunotherapy Using Faeces From Clincal Responders | Melanoma;  RCC;  Head and Neck Squamous Cell Carcinoma | FMT | Phase 2 | 1.Safety evaluation of FMT in advanced cancer patients  2. Tumor response evaluation | Norway |
| NCT04163289 | Preventing Immune-Related Adverse Events in Renal Cell Carcinoma Patients Treated With Combination Immunotherapy Using Fecal Microbiota Transplantation | RCC | FMT | Phase 1 | Occurence of immune-related colitis associated with ipilimumab/nivolumab treatment | Canada |
| NCT05251389 | Conversion of Unresponsiveness to Immunotherapy by Fecal Microbiota Transplantation in Patients With Metastatic Melanoma: a Randomized Phase Ib/IIa Trial | Melanoma | FMT | Phase 1  Phase 2 | Efficacy, defined as clinical benefit (stable disease (SD), partial response (PR), complete response (CR) | Netherlands |
| NCT05502913 | Fecal Microbiota Transplantation to Improve Efficacy of Immune Checkpoint Inhibitors in Metastatic Lung Cancer | Metastatic Lung Cancer | FMT | Phase 2 | Progression-free Survival (PFS) | Israel |
| NCT04056026 | A Single Dose FMT Infusion From a Healthy Family Donor Via Colonoscopy as an Adjunct to Keytruda for the Benefit of Improving Efficacy of Immunotherapy for Metastatic Mesothelioma | Mesothelioma | FMT | Early Phase 1 | PFS | United States |
| NCT04924374 | Microbiota Transplant in Advanced Lung Cancer Treated With Immunotherapy | Lung Cancer | FMT | Not Applicable | Measure of safety | Spain |
| NCT04264975 | Utilization of Microbiome as Biomarkers and Therapeutics in Immuno-Oncology | Solid Carcinoma | FMT | Not Applicable | Overall Response Rate | Korea |
| NCT04729322 | Pilot Trial of Fecal Microbiota Transplantation and Re-Introduction of Anti-PD-1 Therapy in dMMR Colorectal Adenocarcinoma Anti-PD-1 Non-Responders | CRC | FMT | Phase 2 | Objective response rate (ORR) | United State |
| NCT04130763 | Investigator-initiated Trial of Fecal Microbiota Transplant (FMT) Capsule for Improving the Efficacy of Anti-PD-1 in Patients With PD-1 Resistant Digestive System Cancers | Gastrointestinal System Cancer | FMT | Phase 1 | 1.ORR  2.Rate of abnormal vital signs and laboratory test results  3.The number of adverse events | China |
| NCT03686202 | Feasibility Study of Microbial Ecosystem Therapeutics (MET-4) to Evaluate Effects of Fecal Microbiome in Patients on ImmunOtherapy (MET4-IO) | All Solid Tumors | FMT | Early Phase 1 | 1.Cumulative relative abundance of R associated species at day 12 of MET-4  2.Changes in relative abundance of R associated MET-4 strains between baseline and day 12  3.Number of participants with irAEs assessed by CTCAE v.5.0 | Canada |
| NCT05008861 | Safety of Gut Microbiota Reconstruction Plus PD-1/PD-L1 Monoclonal Antibodies to Treat Locally Advanced or Metastatic Non-Small Cell Lung Cancer | NSCLC | FMT | Phase 1 | 1.Incidence of FMT-related Adverse Events  2.Incidence of anti-PD-1/PD-L1-related Adverse Events | China |
| NCT04758507 | Targeting Gut Microbiota to Improve Efficacy of Immune Checkpoint Inhibitors in Patients With Advanced Renal Cell Carcinoma | RCC | FMT | Phase 1  Phase 2 | Number of participants who will be free from tumor progression, as assessed by RECIST criteria v. 1.1. | Italy |
| NCT05273255 | An Open Label Feasibility Study of Fecal Microbiota Transplantation (FMT) in Patients With Malignancies Not Responding to Immune Checkpoint Inhibitor (ICI) Therapy | Cancer | FMT | Not Applicable | Change in the intestinal microbiome community | Switzerland |
| NCT04988841 | Prospective randomIzed Clinical Trial Assessing the Tolerance and Clinical Benefit of feCAl tranSplantation in patientS With melanOma Treated With CTLA-4 and PD-1 Inhibitors | Melanoma | FMT | Phase 2 | Safety | France |
| **Dietary regulation in cancer immunotherapy** | | | | | | |
| NCT05356182 | A Pilot and Feasibility Study of a Dietary Intervention With Low-protein Meals in Cancer Patients Receiving Immunotherapies | Cancer | Diet | Not Applicable | To assess the feasibility of low-protein diet intervention in patients | United States |
| NCT04645680 | Diet and Immune Effects Trial: DIET- A Randomized Double Blinded Dietary Intervention Study in Patients With Metastatic Melanoma Receiving Immunotherapy | Melanoma | Diet | Phase 2 | Change in the gut microbiome | United States |
| NCT05384873 | Multicentre, Randomised, Open-label, Parallel-group Trial to Evaluate Immunonutrition in Improving Efficacy of Immunotherapy in Patients With Metastatic Non-small Cell Lung Cancer, Undergoing Systematic Nutritional Counseling | NSCLC | Dietary Supplement: Immunonutrition | Not Applicable | PFS | Italy |
| NCT03700437 | Randomized Controlled Pilot Study to Evaluate Fasting-mimicking Diet in Patients Receiving Chemo-immunotherapy for Treatment of Metastatic Non-small Cell Lung Cancer | NSCLC | Fasting-Mimicking Diet | Not Applicable | Proportion of the patients who can finish the FMD without serious adverse events | United States |
| NCT04645680 | Diet and Immune Effects Trial: DIET- A Randomized Double Blinded Dietary Intervention Study in Patients With Metastatic Melanoma Receiving Immunotherapy | Melanoma | high-fiber diet | Not Applicable | Change in the gut microbiome | United States |
| NCT04316520 | A Pilot Study Evaluating the Tolerability of a Ketogenic Diet With Vitamin Supplementation for Patients Receiving First Line Treatment for Metastatic Renal Cell Carcinoma | Metastatic Renal Cancer | Ketogenic diet | Not Applicable | Tolerance of one year of ketogenic diet | France |
| **Probiotics and prebiotics application in cancer immunotherapy** | | | | | | |
| NCT04699721 | Clinical Study of Neoadjuvant Chemotherapy and Immunotherapy Combined With Probiotics in Patients With Potential/Resectable Non-small Cell Lung Cancer | NSCLC | *Bifidobacterium trifidum* live powder (BiFico) | Phase 1 | 1.adverse effect  2.Surgical complications  3.non-R0 surgical events | China |
| NCT05032014 | Probiotics Enhance the Treatment of PD-1 Inhibitors in Patients With Liver Cancer | Liver Cancer | *Lactobacillus rhamnosus* Probio-M9 | Not Applicable | ORR | China |
| NCT05094167 | The Mechanism of Probiotic Lactobacillus Bifidobacterium V9(Kex02)Improving the Efficacy of Carilizumab Combined With Platinum in Non-small Cell Lung Cancer Patients | NSCLC | *Lactobacillus Bifidobacterium* V9(Kex02) | Not Applicable | ORR | China |
| NCT05220124 | An Open Label, Randomized Control Study of Probiotics Administration in the Immunotherapy of Urothelial Bladder Carcinoma | Bladder Urothelial Carcinoma | *Bifidobacterium*,*Lactobacillus* and *Enterococcus* Capsules | Phase 4 | PFS | China |
| NCT03870607 | A Randomized Phase II Study of the Administration of Prebiotics and Probiotics During Definitive Treatment With Chemotherapy-radiotherapy for Patients With Squamous Cell Carcinoma of the Anal Canal (BISQUIT) | Anal Cancer Squamous Cell | Prebiotics in combination with probiotics | Phase 2 | Response rate | Brazil |
| NCT03829111 | Pilot Study to Evaluate the Biologic Effect of CBM588 in Combination With Nivolumab/Ipilimumab for Patients With Metastatic Renal Cell Carcinoma | RCC | *Clostridium* *butyricum* (CBM) 588 | Phase 1 | Change in *Bifidobacterium* composition of stool | United States |
